# JAML promotes acute kidney injury mainly through a macrophage-dependent mechanism

**DOI:** 10.1172/jci.insight.158571

**Published:** 2022-06-16

**Authors:** Wei Huang, Bi-Ou Wang, Yun-Feng Hou, Yi Fu, Si-Jia Cui, Jing-Han Zhu, Xin-Yu Zhan, Rong-Kun Li, Wei Tang, Ji-Chao Wu, Zi-Ying Wang, Mei Wang, Xiao-Jie Wang, Yan Zhang, Min Liu, Yu-Sheng Xie, Yu Sun, Fan Yi

**Affiliations:** 1 Key Laboratory of Infection and Immunity of Shandong Province, Department of Pharmacology, School of Basic Medical Sciences, Shandong University, Jinan, China.; 2 Intensive Care Unit, Shandong Provincial Qianfoshan Hospital, the First Affiliated Hospital of Shandong First Medical University, Jinan, China.; 3 Department of Pathogenic Biology, School of Basic Medical Sciences, Shandong University, Jinan, China.; 4 Key Laboratory of Cardiovascular Remodeling and Function Research, Chinese Ministry of Education and Chinese Ministry of Health, and State and Shandong Province Joint Key Laboratory of Translational Cardiovascular Medicine, Qilu Hospital, Shandong University, Jinan, China.

**Keywords:** Nephrology, Macrophages

## Abstract

Although macrophages are undoubtedly attractive therapeutic targets for acute kidney injury (AKI) because of their critical roles in renal inflammation and repair, the underlying mechanisms of macrophage phenotype switching and efferocytosis in the regulation of inflammatory responses during AKI are still largely unclear. The present study elucidated the role of junctional adhesion molecule–like protein (JAML) in the pathogenesis of AKI. We found that JAML was significantly upregulated in kidneys from 2 different murine AKI models including renal ischemia/reperfusion injury (IRI) and cisplatin-induced AKI. By generation of bone marrow chimeric mice, macrophage-specific and tubular cell–specific *Jaml* conditional knockout mice, we demonstrated JAML promoted AKI mainly via a macrophage-dependent mechanism and found that JAML-mediated macrophage phenotype polarization and efferocytosis is one of the critical signal transduction pathways linking inflammatory responses to AKI. Mechanistically, the effects of JAML on the regulation of macrophages were, at least in part, associated with a macrophage-inducible C-type lectin–dependent mechanism. Collectively, our studies explore for the first time to our knowledge new biological functions of JAML in macrophages and conclude that JAML is an important mediator and biomarker of AKI. Pharmacological targeting of JAML-mediated signaling pathways at multiple levels may provide a novel therapeutic strategy for patients with AKI.

## Graphical abstract

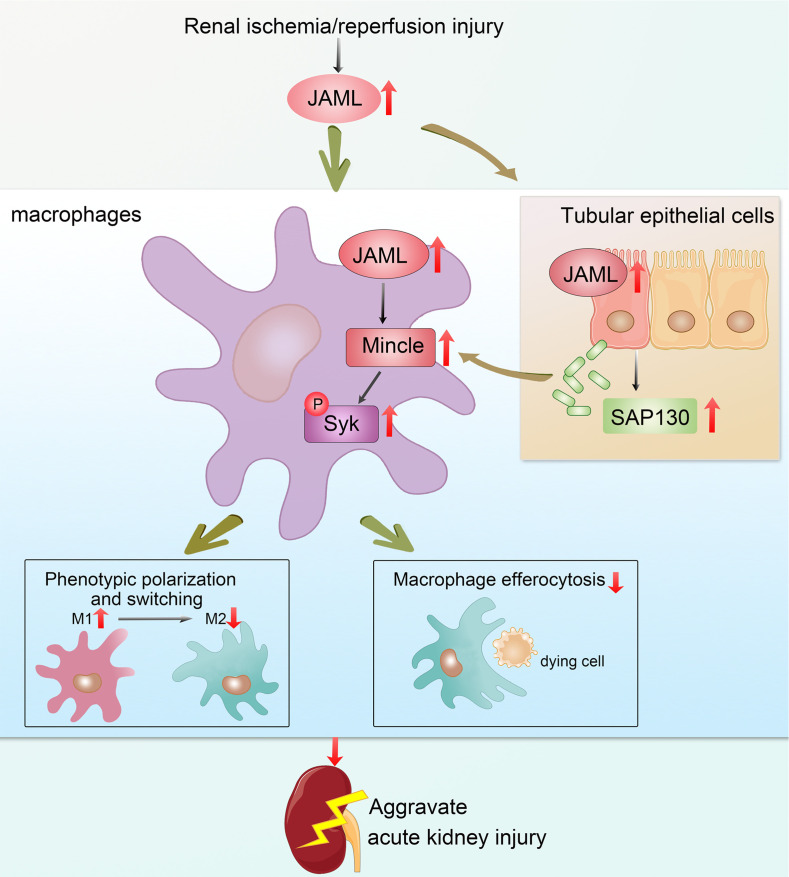

## Introduction

Acute kidney injury (AKI), often caused by renal ischemia/reperfusion injury (IRI), nephrotoxic agents, and sepsis, is a global public health concern associated with high morbidity and mortality (1, 2). Currently, other than dialysis, no therapeutic interventions reliably improve survival, limit injury, or speed recovery after AKI (3). Numerous studies have demonstrated that the inflammatory response plays a vital role in the pathogenesis of various AKIs (4–6). Thus, better understanding of the cellular and molecular mechanisms underlying the inflammatory response has high potential for identifying effective therapies to prevent or ameliorate AKI. During AKI, a complex network of interactions among recruited immune cells, resident immune cells, and renal parenchymal cells sets major innate immunity pathways in motion by activating pattern recognition receptors (PRRs) and releasing various inflammatory mediators (7–10). Among inflammatory myeloid cells, macrophages are undoubtedly attractive therapeutic targets for AKI because of their critical roles in renal inflammation and repair (11–15). Macrophages are highly heterogeneous and their differentiation into morphologically and functionally distinct phenotypes M1/M2 depends on the microenvironment and the nature or the stage of disease (16). Inflammatory M1 macrophages are recruited to the kidney, where they amplify inflammatory responses and promote parenchymal injury. In contrast, M2 macrophages show antiinflammatory effects after AKI and facilitate renal repair (17). In the process of inflammation resolution, a process termed “efferocytosis” for the clearance of apoptotic or dying cells by macrophages is also essential for maintaining tissue homeostasis (18, 19). Accordingly, defective efferocytosis exacerbates autoimmune diseases and sterile inflammation (20, 21). However, the underlying mechanisms of M1 and M2 macrophage phenotype switching and efferocytosis in the regulation of inflammatory responses during AKI are still largely unclear. Therefore, identification of key factors controlling the phenotype and functions of macrophages may offer a new avenue for the development of therapeutic strategies for patients with AKI.

Recently, the role of junctional adhesion molecules (JAMs) of the immunoglobulin superfamily has attracted much attention because of their important functions in immune cell activation and inflammatory responses (22, 23). JAMs are expressed by leukocytes, platelets, and epithelial and endothelial cells and play critical roles in the regulation of cell polarity, epithelial barrier formation, and leukocyte migration (22–25). Junctional adhesion molecule–like protein (JAML), as a novel identified member of the JAM family, is expressed on a variety of effector cells of both innate and adaptive immunity, including monocytes, neutrophils, and memory T cells, as well as some parenchymal cells (26–29). Emerging evidence has revealed that JAML is vital to regulate leukocytes’ adhesion and transendothelial migration and is a costimulatory receptor for epithelial γδ T cell activation (26, 27, 30, 31), indicating the potential role of JAML in triggering inflammation and tissue repair. However, JAML’s contribution to AKI and mechanism of regulation of macrophage dynamics and functions are still unknown. In this study, we explored the potentially new biological functions of JAML in promoting AKI and found that JAML-mediated macrophage phenotype polarization and efferocytosis is one of the critical signal transduction pathways linking inflammatory responses to AKI, suggesting that JAML may be an attractive therapeutic target and biomarker for AKI.

## Results

### JAML is upregulated in kidneys from patients with AKI and mice with renal IRI.

**Figure 1:**
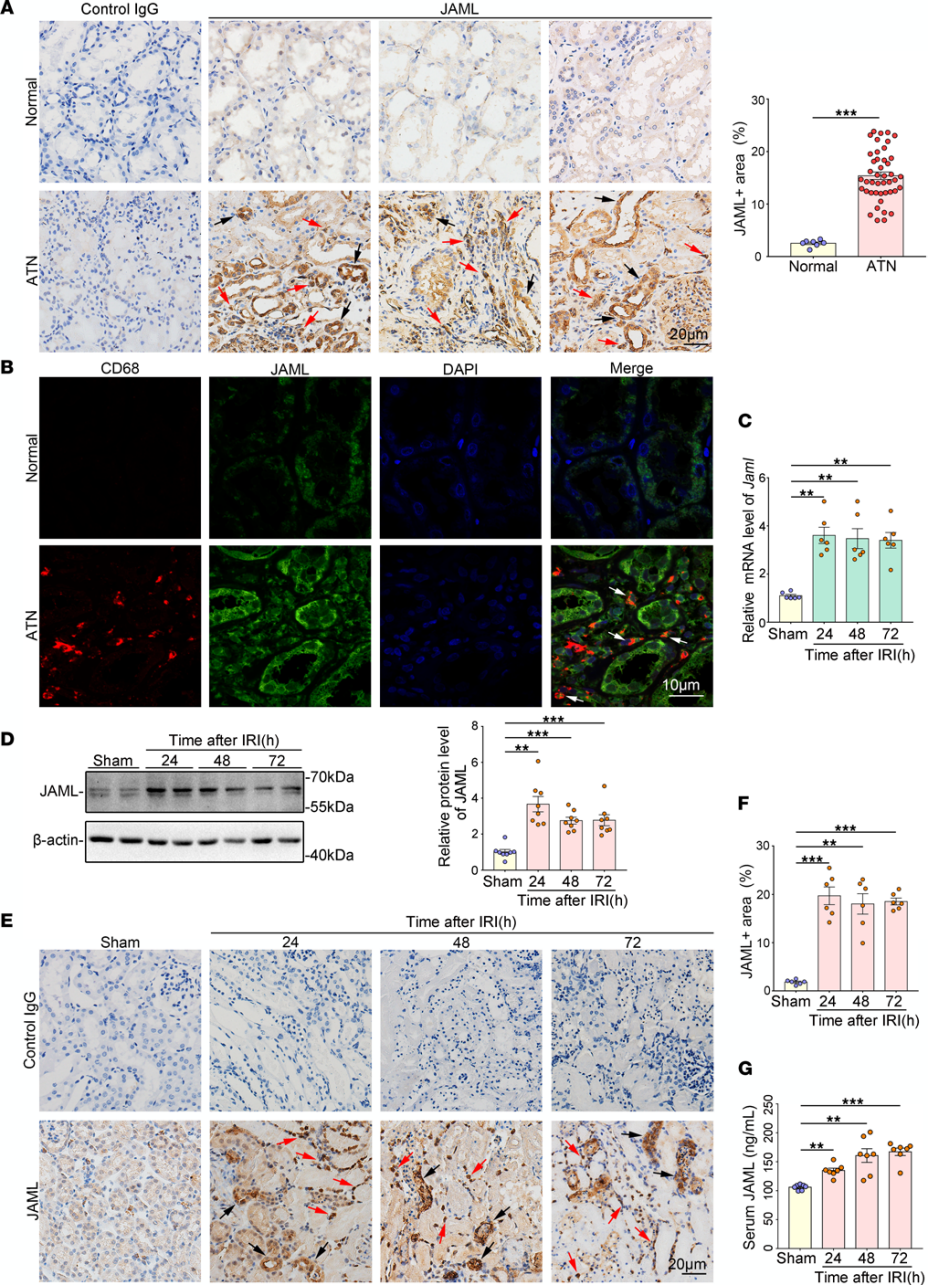
JAML is upregulated in kidneys from patients with AKI and mice with renal IRI. (**A**) Representative IHC images and quantification of JAML in kidneys from human normal kidney poles (*n* = 7) and patients with biopsy-proven acute tubular necrosis (ATN) (*n* = 44). Red arrows indicate representative positive cells in renal interstitium; black arrows indicate renal parenchymal cells. Human kidneys stained with normal IgG in place of the corresponding primary antibodies were a negative control. Scale bar: 20 μm. (**B**) Representative fluorescence multiplexed IHC images of JAML (green) and CD68 (red) in kidneys from human normal kidney poles and patients with ATN (*n* = 6). Arrows indicate the expression of JAML in macrophages in renal interstitium. Scale bar: 10 μm. (**C**) Relative mRNA level of *Jaml* in kidneys from mice with renal IRI (*n* = 6). (**D**) Representative Western blot and quantifications of JAML expression in kidneys from mice with renal IRI (*n* = 8). (**E**) Representative IHC images and (**F**) quantification of JAML in kidneys from mice with renal IRI (*n* = 6). Red and black arrows indicate the same as in **A**. Scale bar: 20 μm. (**G**) Serum level of JAML in mice with renal IRI (*n* = 7). Data are shown as mean ± SEM. **P < 0.01, ***P < 0.001. Two-tailed Student’s unpaired *t* test (**A**), 1-way ANOVA test (**C**, **D**, **F**, and **G**). See complete unedited blots in the supplemental material.

By immunohistochemical (IHC) staining analysis (Figure 1A), we first observed the upregulation of JAML in kidneys from patients with biopsy-proven acute tubular necrosis (ATN), which is one of the most common causes of AKI. We also found that JAML was not only localized in the renal interstitium but also expressed and induced in renal parenchymal cells after AKI (Figure 1A). By fluorescence multiplexed IHC consecutive staining on a single slide, we further determined JAML expression in macrophages in the interstitium (Figure 1B). In mice with renal IRI, JAML expression was elevated in the kidney after 30 minutes of renal ischemia followed by different time points of reperfusion in mice by mRNA (Figure 1C), Western blot (Figure 1D), and IHC (Figure 1, E and F) analyses. In addition, the serum level of JAML was also enhanced compared with controls (Figure 1G).

### JAML deficiency protects against renal injury and alleviates inflammatory responses in mice with renal IRI.

By generation of global *Jaml*-knockout (*Jaml*^–/–^) mice, which was confirmed by mRNA (Supplemental Figure 1A; supplemental material available online with this article; https://doi.org/10.1172/jci.insight.158571DS1), Western blot (Supplemental Figure 1B), and IHC staining (Supplemental Figure 1C) analyses in the kidney, we found that JAML deficiency significantly reduced renal IRI in mice as evidenced by decreased levels of serum creatinine (Supplemental Figure 1D) and blood urea nitrogen (Supplemental Figure 1E). As shown in Supplemental Figure 1F, in parallel with improved morphological injuries and reduced dying cells, the level of kidney injury molecule 1 (KIM-1), which is an early biomarker of AKI and correlated with kidney tissue damage, was also significantly reduced in *Jaml*^–/–^ mice compared with wild-type (WT) mice with renal IRI. Furthermore, JAML deficiency attenuated inflammatory responses by decreasing macrophage and neutrophil infiltration (Supplemental Figure 2A), the levels of proinflammatory mediators (Supplemental Figure 2, B–E) in the kidney from mice with renal IRI.

### JAML in bone marrow–derived immune cells primarily contributes to renal IRI.

**Figure 2:**
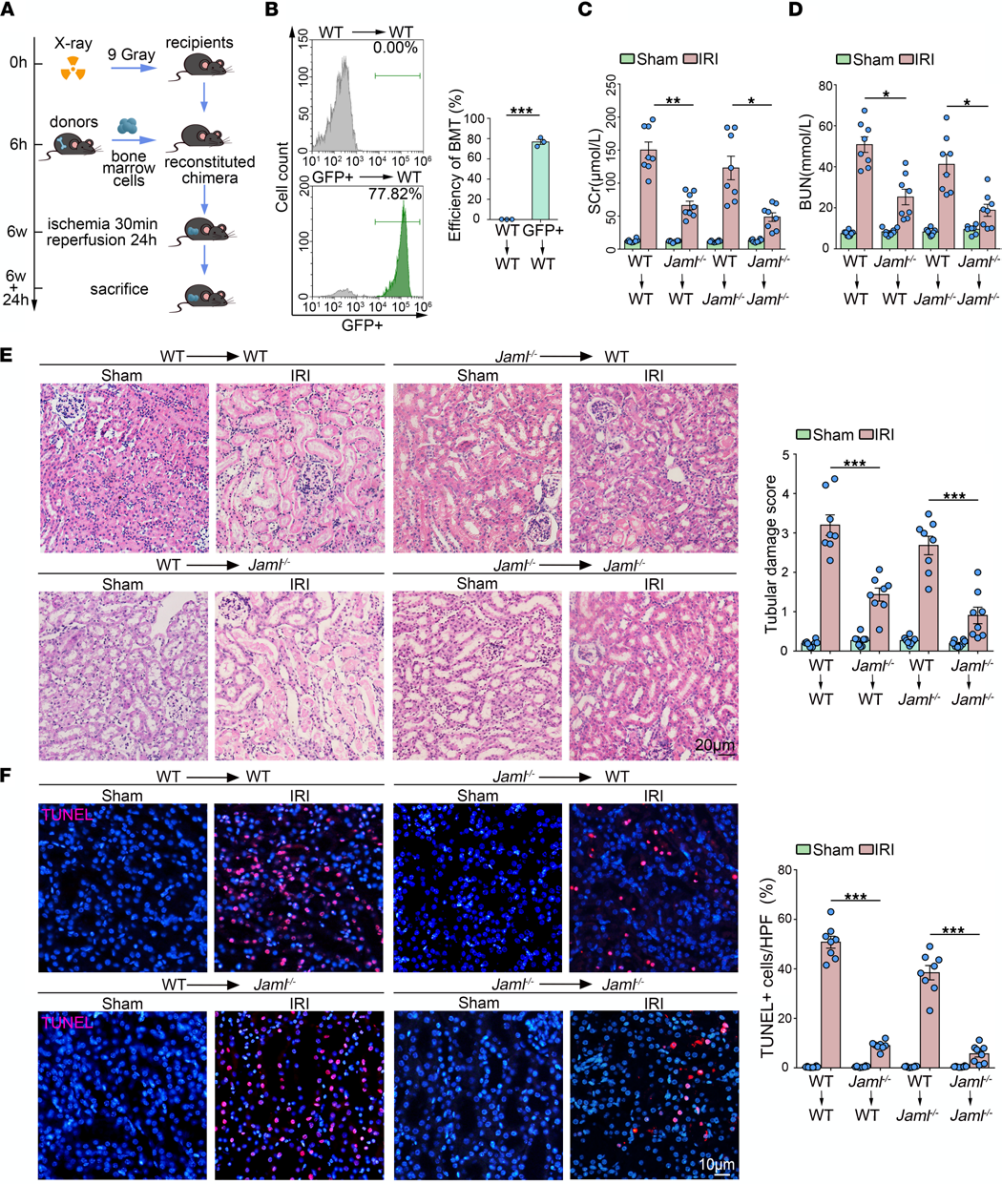
JAML in BM-derived immune cells primarily contributes to renal IRI. (**A**) A schematic diagram showing the procedure of BM transplantation for experimental mice. (**B**) Representative GFP expression profile of recipient BM cells 6 weeks after BM transplantation by flow cytometry analysis. The proportion of GFP^+^ cells in the reconstructed BM was about 80% (*n* = 3). (**C**) Serum creatinine (SCr) concentration in different groups of mice after renal IRI (*n* = 8). (**D**) Blood urea nitrogen (BUN) levels of different groups of mice after renal IRI (*n* = 8). (**E**) Representative images of H&E staining showing the morphology of kidney and quantitative assessment of tubular damage in the kidney from different groups of mice (*n* = 8). Scale bar: 20 μm. (**F**) In situ TUNEL assays and quantification were performed to assess renal cell death in the kidney from different groups of mice (*n* = 8). Scale bar: 10 μm. Data are mean ± SEM. *P < 0.05, **P < 0.01, ***P < 0.001. Two-tailed Student’s unpaired *t* test (**B**), 2-way ANOVA test (**C**–**F**).

Considering that JAML was expressed not only in immune cells such as macrophages but also in renal parenchymal cells, we next determined JAML’s contribution in bone marrow–derived (BM-derived) immune cells or renal parenchymal cells individually to the pathogenesis of renal IRI through BM transplantation studies. Chimeric mice were created, in which BM was replaced with donor BM cells from WT or from *Jaml^–/–^* mice. Six weeks after BM transplantation, chimeric mice were subjected to renal IRI (Figure 2A). Meanwhile, by using GFP-transgenic mice as donors, BM reconstitution in the recipient mice was confirmed by flow cytometry analysis of GFP expression. The proportion of GFP^+^ cells in the reconstructed BM was about 80% (Figure 2B). WT→ WT chimeras developed renal injury after IRI as assessed by elevated serum creatinine (SCr, Figure 2C), blood urea nitrogen (BUN, Figure 2D), and tubular damage (Figure 2E and Supplemental Figure 3), as well as displaying an increase in renal cell death (Figure 2F). Notably, in comparison with WT→ WT chimeras, *Jaml*^–/–^→ WT chimeras that lacked myeloid JAML significantly ameliorated renal IRI. Similar results were also found in *Jaml*^–/–^→ *Jaml*^–/–^ chimeras compared with WT→ *Jaml*^–/–^ mice. However, neither WT→ *Jaml*^–/–^ nor *Jaml*^–/–^→ *Jaml*^–/–^ mice (lacking renal parenchymal JAML in recipient mice) had obvious damage reduction compared to WT→ WT or *Jaml*^–/–^→ WT chimeras, respectively, although a trend toward reduction was observed. Thus, the presence of JAML in immune cells worsened renal damage and appears to be a major contributor to AKI.

### Macrophage JAML is required in the pathogenesis of AKI.

**Figure 3:**
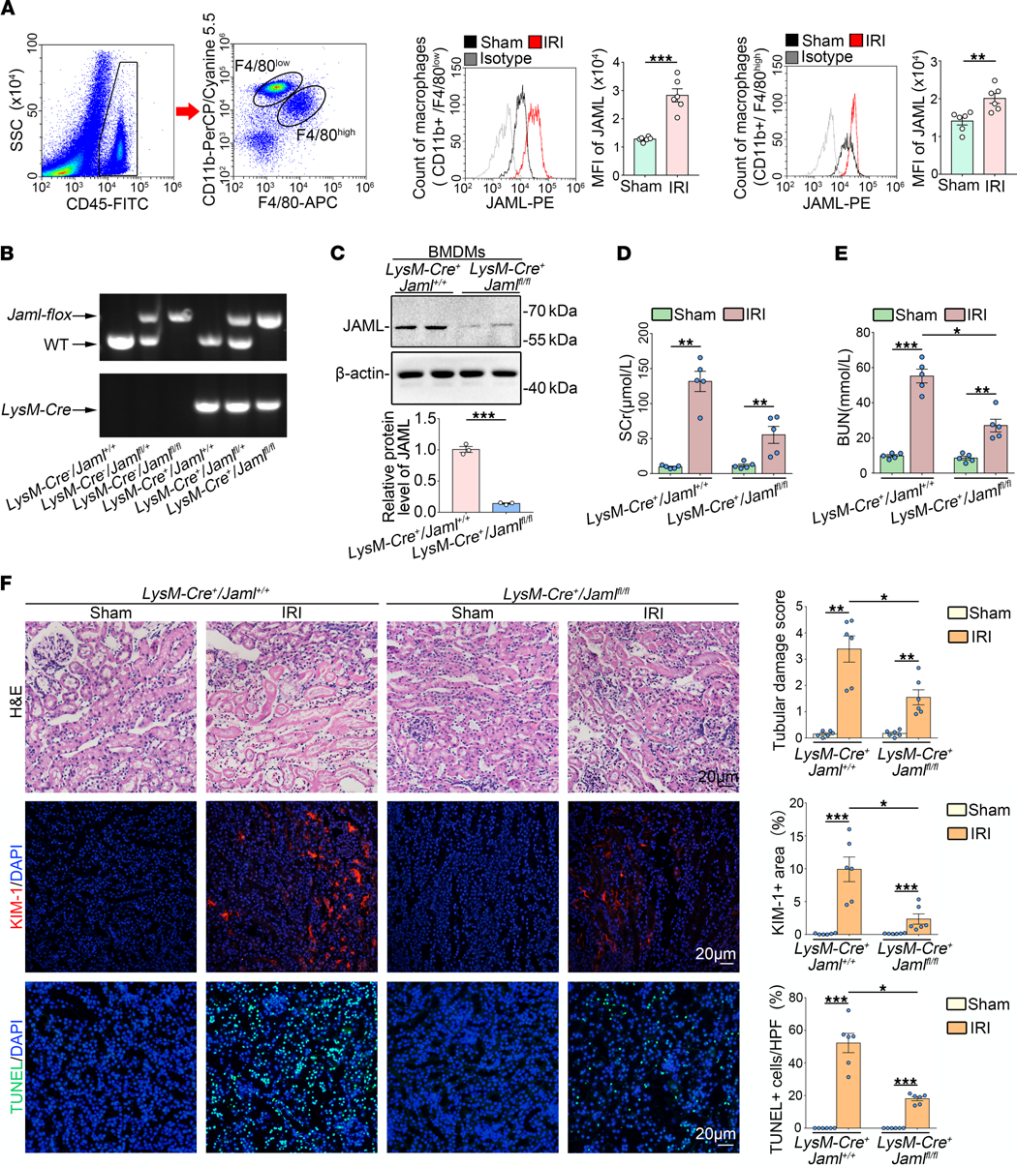
Macrophage JAML is required in the pathogenesis of IRI-induced renal injury. (**A**) Flow cytometry analysis of macrophages freshly isolated from the kidney in mice with renal IRI. CD45-positive cells were divided into F4/80^lo^ and F4/80^hi^ macrophages. Representative histogram showing cell surface JAML expression on 2 subsets of macrophages and quantitative analysis of the MFI of JAML-phycoerythrin (*n* = 6). (**B**) Genotyping was confirmed by tail preparation and PCR at 2 weeks of age (*n* = 8). (**C**) Representative Western blot and quantifications of JAML expression in the BM-derived macrophages from *LysM-Cre^+^* *Jaml^fl/fl^* mice (*n* = 3). (**D**) SCr concentration in different groups of mice after renal IRI (*n* = 5). (**E**) BUN levels of different groups of mice after renal IRI (*n* = 5). (**F**) Representative images of H&E staining and quantitative assessment of tubular damage in the kidney from different groups of mice (upper). Representative images and quantification of immunofluorescence staining of kidney injury molecule 1 (KIM-1) (red) (middle). In situ TUNEL assays and quantification were performed to assess renal cell death (lower) (*n* = 6). Scale bar: 20 μm. HPF, high power field. Data are mean ± SEM. *P < 0.05, **P < 0.01, ***P < 0.001. Two-tailed Student’s unpaired *t* test (**A** and **C**), 2-way ANOVA test (**D**–**F**). See complete unedited blots in the supplemental material.

Considering that macrophages and neutrophils are predominant myeloid cell types that play a critical role in renal inflammation and repair following AKI (5, 6), we examined JAML expression on macrophages and neutrophils isolated from the kidneys of mice with renal IRI by flow cytometry analysis. For macrophages, 2 subtypes were further identified based on the F4/80 and CD11b fluorescence intensity (32–37). Infiltrating macrophages, which arrive to the kidney from the peripheral blood, are CD11b^hi^ F4/80^lo^ (F4/80^lo^). Resident macrophages, which are largely embryonically derived, are CD11b^lo^F4/80^hi^ (F4/80^hi^) (Figure 3A). Then, gating on F4/80^lo^ and F4/80^hi^ populations of macrophages from the kidney in mice with renal IRI and their controls, we found that JAML was more significantly upregulated on F4/80^lo^ macrophages from the injured kidney compared with F4/80^hi^ populations as shown by MFI (Figure 3A). However, no appreciable changes of JAML expression were observed on neutrophils (Supplemental Figure 4A).

Moreover, we generated macrophage-specific *Jaml*-knockout (*LysM-Cre^+^* *Jaml^fl/fl^*) mice by intercrossing *Jaml*-floxed mice with *LysM* (*Lyz2)-Cre* mice (Figure 3B and Supplemental Figure 4B). BMDMs isolated from *LysM-Cre^+^* *Jaml^fl/fl^* mice had an almost complete absence of JAML expression (Figure 3C). Consistently, *LysM-Cre^+^ Jaml^fl/fl^* mice following renal IRI exhibited obvious improvement in renal function (Figure 3, D and E), tubular injury, and cell death (Figure 3F) in the kidney as compared with *LysM*-*Cre^+^* *Jaml^+/+^* mice, further confirming the pathological significance of macrophage JAML in AKI.

### Renal tubule–specific JAML deletion in mice only slightly ameliorates renal IRI.

**Figure 4:**
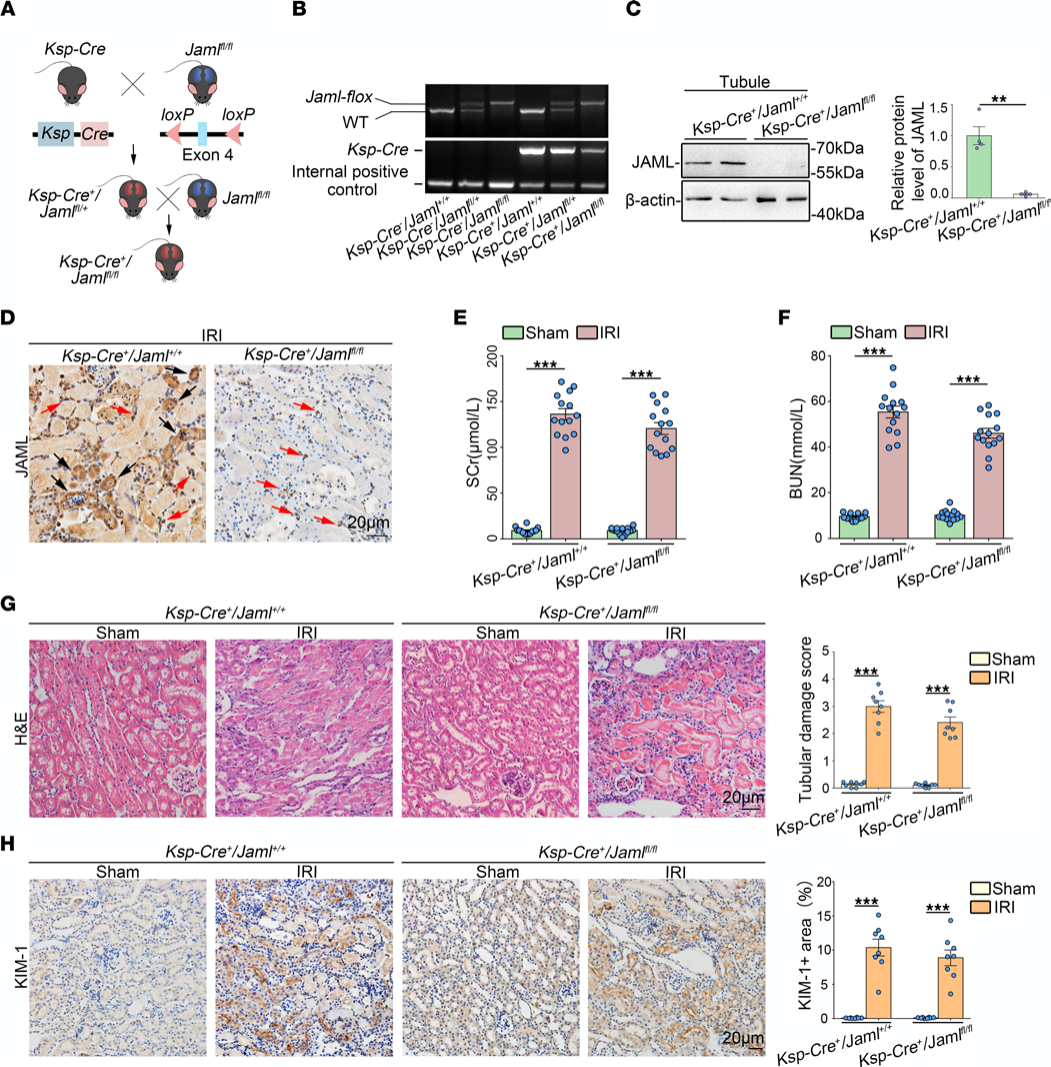
Tubule-specific JAML deletion in mice only slightly ameliorates renal IRI. (**A** and **B**) Experimental scheme for generating conditional knockout mice in which *Jaml* is specifically ablated in renal tubular cells (*Ksp-Cre^+^* *Jaml^fl/fl^*) by using *Cre-loxP* recombination system. Exon 4 is deleted upon *Ksp-Cre*–mediated recombination (**A**). Genotyping was confirmed by tail preparation and PCR at 2 weeks of age (*n* = 8) (**B**). (**C**) Representative Western blot and quantification of JAML expression in isolated tubules from *Ksp-Cre^+^* *Jaml^+/+^* and *Ksp-Cre^+^* *Jaml^fl/fl^* mice (*n* = 4). (**D**) Representative IHC images of JAML in kidneys from *Ksp-Cre^+^ Jaml^+/+^* and *Ksp-Cre^+^* *Jaml^fl/fl^* mice with IRI (*n* = 5). Red arrows indicate representative JAML positive interstitial cells; black arrows indicate renal parenchymal cells. (**E**) SCr concentration in different groups of mice (*n* = 14). (**F**) BUN levels of different groups of mice (*n* = 14). (**G**) Representative images of H&E staining and quantitative assessment of tubular damage in the kidney from different groups of mice (*n* = 8). (**H**) Representative images and quantifications of IHC staining of KIM-1 in the kidney from different groups of mice (*n* = 8). Scale bar: 20 μm. Data are mean ± SEM. **P < 0.01, ***P < 0.001. Two-way ANOVA test (**E**–**H**), 2-tailed Student’s unpaired *t* test analysis (**C**). See complete unedited blots in the supplemental material.

We also generated renal tubule–specific knockout mice (*Ksp*-*Cre^+^ Jaml^fl/fl^*, Figure 4A), which was confirmed by tail genotyping (Figure 4B), Western blot analysis of isolated tubules (Figure 4C), and IHC staining in the kidney (Figure 4D), to further examine the contribution of JAML in renal tubular cells to renal IRI. *Ksp*-*Cre^+^ Jaml^fl/fl^* mice were phenotypically normal and had no appreciable defect in renal morphology and function compared to *Ksp*-*Cre^+^ Jaml^+/+^* littermates. However, *Ksp*-*Cre^+^ Jaml^fl/fl^* mice following renal IRI exhibited a trend of improvements in renal function (Figure 4, E and F), tubular injury (Figure 4, G and H), and cell death (Supplemental Figure 5) in the kidney as compared with *Ksp*-*Cre^+^* *Jaml^+/+^* mice but no significant difference.

### JAML facilitates macrophage-inducible C-type lectin signaling.

**Figure 5:**
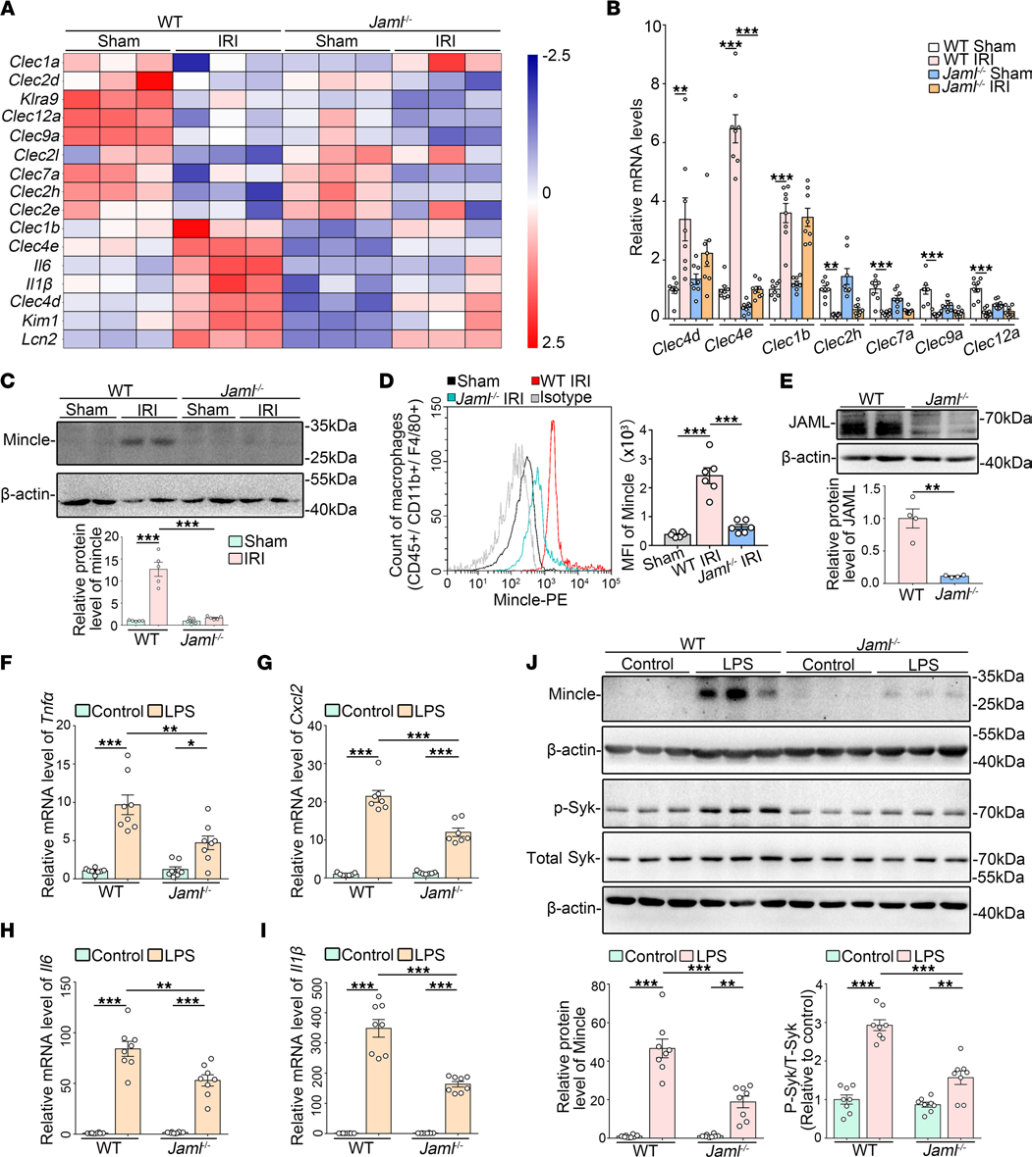
Upregulation of JAML facilitates macrophage-inducible C-type lectin signaling. (**A**) Representative heatmap of gene expression levels in the kidney from different groups of mice with IRI by microarray analysis (*n* = 3). (**B**) Relative mRNA levels of C-type lectin members including *Clec4d*, *Clec4e* (macrophage-inducible C-type lectin, Mincle), *Clec1b*, *Clec2h*, *Clec7a*, *Clec9a*, and *Clec12a* in kidneys from different groups of mice (*n* = 8). (**C**) Representative Western blots and quantifications of Mincle in kidneys from different groups of mice (*n* = 5). (**D**) Representative flow cytometry histogram showing cell surface Mincle expression on macrophages freshly isolated from the kidney in different groups of mice and quantitative analysis of MFI of Mincle-phycoerythrin (*n* = 6). (**E**) Representative Western blot and quantifications of JAML expression in BMDMs from WT or *Jaml*^–/–^ mice (*n* = 4). (**F**–**I**) Relative mRNA levels of proinflammatory mediators including *Tnfα* (**F**), *Cxcl2* (**G**), *Il6* (**H**), and *Il1β* (**I**) in LPS-treated BMDMs (*n* = 8). (**J**) Representative Western blots and quantifications of Mincle and phosphorylated and total spleen tyrosine kinase (Syk) in different groups of BMDMs (*n* = 8). Data are mean ± SEM. *P < 0.05, **P < 0.01, ***P < 0.001. Two-tailed Student’s unpaired *t* test (**E**), 2-way ANOVA test (**C**, **D**, **F**–**J**). See complete unedited blots in the supplemental material.

By microarray analysis of global gene expression in kidneys from *Jaml*^–/–^ mice with renal IRI, we found notable changes in some members of C-type lectin receptors (CLRs), which belong to a family of PRRs (Figure 5A). Real-time reverse transcription PCR (RT-PCR) (Figure 5B) and Western blot (Figure 5C) analyses further verified that JAML deficiency markedly attenuated IRI-induced Mincle (also called *Clec4e*) expression in the kidney but had no significant effects on other CLRs, such as *Clec4d*, *Clec1b*, *Clec2h*, *Clec7a*, *Clec9a*, and *Clec12a* (Figure 5B). Consistent with previous studies showing that Mincle is selectively expressed and induced in macrophages (38) after AKI, we found that Mincle expression was obviously lower in macrophages isolated from the kidneys of *Jaml*^–/–^ mice compared with that from WT mice by flow cytometry analysis (Figure 5D). Consistently, by IHC staining analysis (Supplemental Figure 6A), Mincle expression was markedly decreased in the interstitium of injured kidneys in *LysM-Cre^+^ Jaml^fl/fl^* mice relative to *LysM*-*Cre^+^* *Jaml^+/+^* mice.

To elucidate the role of JAML in macrophages, we first treated BMDMs with LPS and confirmed the enhanced expression of JAML on macrophages in vitro (Supplemental Figure 6B). JAML deficiency on BMDMs isolated from *Jaml*^–/–^ mice (Figure 5E) alleviated inflammatory responses under LPS treatment (Figure 5, F–I). Consistent with in vivo results, Mincle expression significantly changed as assessed by mRNA (Supplemental Figure 6C) and Western blot analyses (Figure 5J) in BMDMs. Moreover, JAML deficiency inhibited the activation of Syk, a well-recognized direct downstream effector of Mincle (Figure 5J), which was reversed by Mincle overexpression (Supplemental Figure 6, D and E).

### JAML regulates macrophage phenotypic polarization and efferocytosis via a Mincle-dependent mechanism.

**Figure 6:**
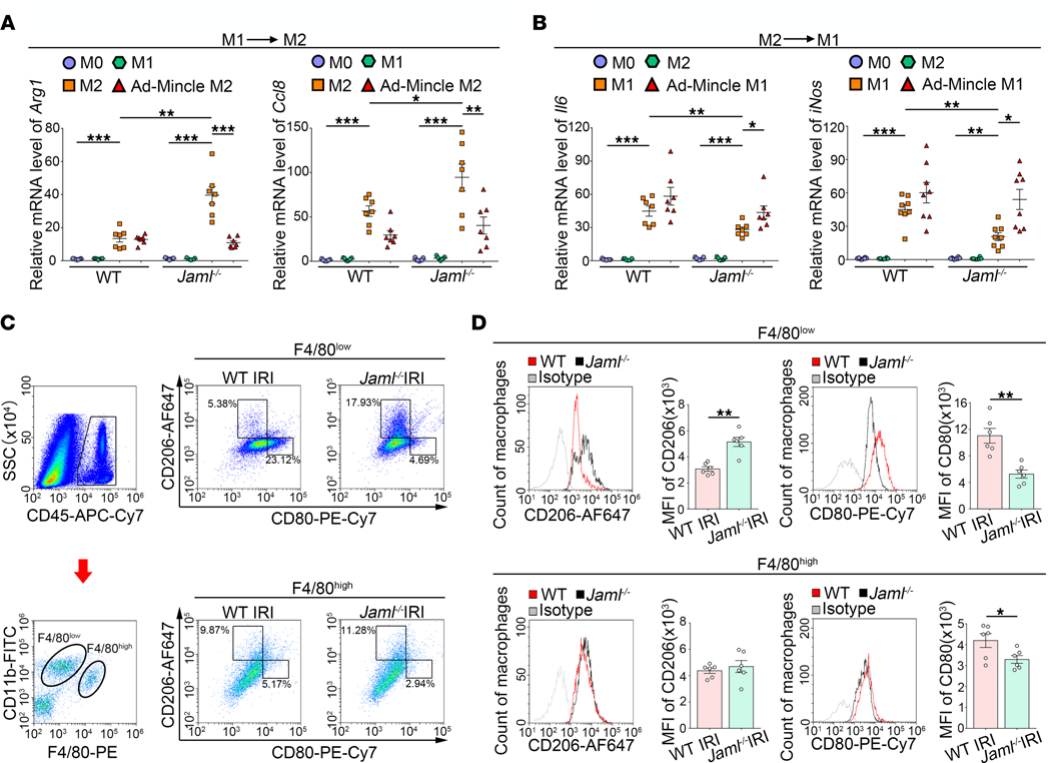
JAML regulates macrophage phenotypic polarization via a Mincle-dependent mechanism. (**A** and **B**) BMDMs from WT or *Jaml*^–/–^ mice were serum starved for 4 hours (M0) and then polarized to M1 (LPS) or M2 (IL-4) for 8 hours. Media were removed, M1 macrophages were treated with M2 stimuli (IL-4), and M2 macrophages were treated with M1 stimuli (LPS) for an additional 8 hours. Real-time PCR was performed to measure *Arg1* and *Ccl8* expression in M1 macrophages polarized to M2 (**A**). *Il6* and *iNos* gene expression was measured in M2 macrophages polarized to M1 (**B**) (*n* = 7). (**C**) Flow cytometry analysis of renal macrophages in the injured kidney after IRI. CD45-positive cells were divided into the F4/80^lo^ and F4/80^hi^ groups. Representative flow cytometry analysis of M1 (CD80^hi^) and M2 (CD206^hi^) cell populations in F4/80^lo^ and F4/80^hi^ macrophages isolated from the kidney in different groups of mice. SSC, side scatter. (**D**) Representative flow cytometry histogram showing cell surface marker CD206 (M2) and CD80 (M1) expression on 2 subtypes of macrophages and quantitative analysis of MFI of CD206-AF647 or CD80-PE-Cy7 (*n* = 6). Data are mean ± SEM. *P < 0.05, **P < 0.01, ***P < 0.001. Two-way ANOVA test (**A** and **B**), 2-tailed Student’s unpaired *t* test (**D**).

Next, we polarized BMDMs isolated from WT or *Jaml*^–/–^ mice into M1 or M2 macrophages, before switching them back into M2 or M1 macrophages, respectively. A panel of specific M2 or M1 marker genes was quantified by real-time RT-PCR. In the absence of JAML, M1 macrophages that had been switched to M2 showed significantly enhanced expressions of M2-associated genes *Arg1* and *Ccl8* (Figure 6A), while JAML-deficient M2 macrophages that had been switched to M1 showed lower expression of M1-related genes *Il6* and *iNos* (Figure 6B). All these changes were counteracted by Mincle overexpression (Figure 6, A and B). Similar results were verified by ELISA (Supplemental Figure 7A).

We also tried to assess the effect of JAML on macrophage polarization in vivo. As previous studies described (39, 40), we detected the polarization status of 2 subtypes of macrophages, F4/80^lo^ (infiltrating) and F4/80^hi^ (resident), in the mouse kidney by flow cytometry analysis. A significantly higher proportion of M2 (CD206^hi^) or lower proportion of M1 (CD80^hi^) macrophages was found in *Jaml^–/–^* IRI mice than in control mice (Figure 6C and Supplemental Figure 7B). In addition, the MFI of CD80 or CD86, 2 markers of M1-like macrophages, was markedly decreased, while CD206 (M2 marker) was greater in *Jaml*^–/–^ mice than in WT littermates after renal IRI. Of note, JAML deficiency promoted phenotypic polarization toward M2, which was much more apparent in infiltrating macrophages than that in resident macrophages (Figure 6D and Supplemental Figure 7C).

**Figure 7:**
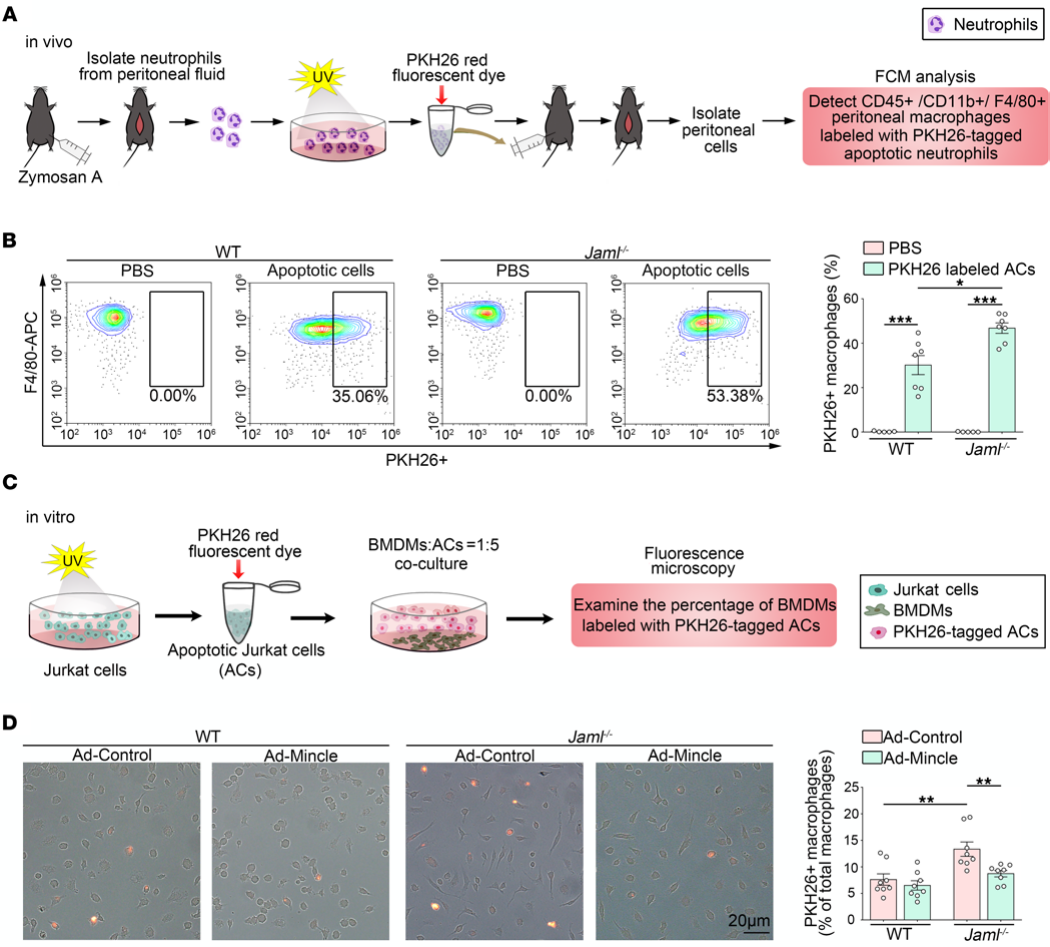
JAML regulates macrophage efferocytosis via a Mincle-dependent mechanism. (**A**) Schematic diagrams showing the procedure of macrophage efferocytosis assay in vivo. FCM, flow cytometry. (**B**) Mice were injected intraperitoneally with PKH26-labeled apoptotic cells, and 45 minutes later lavage fluid was analyzed by flow cytometry for the percentage of F4/80^+^ macrophages that had incorporated the labeled neutrophils (*n* = 7). (**C**) Schematic diagrams showing the procedure of macrophage efferocytosis assay in vitro. (**D**) Overlay images of phase and fluorescence microscopy images of cultured BMDMs treated for 2 hours with UV-exposed PKH26-labeled Jurkat cells. Quantitative analysis of percentage of PKH26^+^ macrophages (*n* = 8). Data are mean ± SEM. *P < 0.05, **P < 0.01, ***P < 0.001. Two-way ANOVA test (**B** and **D**).

In addition, we found that JAML/Mincle signaling regulated macrophage efferocytosis. To evaluate the efferocytic capacity of peritoneal macrophages in vivo, we injected the peritoneum with fluorescently labeled apoptotic neutrophils. After 45 minutes, the peritoneal cells were collected and analyzed by flow cytometry (Figure 7A). Uptake of the injected apoptotic neutrophils by F4/80^+^ macrophages in *Jaml*^–/–^ mice was higher than that in WT mice (Figure 7B). Moreover, in vitro, we induced apoptosis in PKH67-labeled Jurkat cells via exposure to UV radiation (Figure 7C) and then administrated the apoptotic cells to BMDMs for 2 hours and washed the cultures to remove any free apoptotic cells. Fluorescence microscopy was used to assess efferocytosis. Our data showed that macrophages from *Jaml*^–/–^ mice had significantly higher efferocytosis than those from WT mice, which was reversed by overexpression of Mincle (Figure 7D).

To further investigate JAML’s modulation of macrophage efferocytosis through Mincle during AKI, we double stained kidney sections in situ with TUNEL reagents and CD68 antibody based on previous studies (41, 42). As expected, the injured kidneys of *Jaml*^–/–^ mice had a higher ratio of macrophage-associated TUNEL^+^ apoptotic cells to free apoptotic cells compared with WT mice, even though the number of macrophages was reduced (Supplemental Figure 7D). Moreover, after Mincle was significantly overexpressed in macrophages from *Jaml*^–/–^ mice as determined by Western blot analysis (Supplemental Figure 7E), adoptive transfer of macrophages was performed in recipient mice with AKI where chemical deletion of macrophages was achieved by clodronate-liposome. We found that the macrophage efferocytosis was markedly enhanced in the injured kidneys of *Jaml^–/–^* mice that received *Jaml*-deficient macrophages compared with control mice. These effects were significantly inhibited by adoptive transfer with Mincle overexpressed *Jaml*-deficient macrophages (Supplemental Figure 7F), suggesting that JAML attenuated macrophage efferocytosis through Mincle during AKI.

### Gene silencing of JAML inhibits the release of endogenous Mincle ligands from proximal tubule epithelial cells.

Given that JAML was upregulated in renal tubules under ischemic conditions and renal tubule deletion of JAML seemed to lead to a slight protective effect, we then detected the role of JAML in NRK-52E cells. We used 3 approaches to mimic hypoxia conditions, including oxygen-glucose deprivation (OGD) (Supplemental Figure 8A), chemical anoxia/recovery induced by incubating cells in glucose-free medium with antimycin A/2-deoxyglucose for ATP depletion (anoxia) and then in glucose-replete complete growth medium (recovery) (Supplemental Figure 8B), or CoCl_2_ treatment (Supplemental Figure 8C). All these manipulations significantly induced JAML expression. Interestingly, although gene silencing of *Jaml* (Supplemental Figure 8D) had no obvious effects on OGD-induced cell apoptosis (Supplemental Figure 8E) and the expression of spliceosome-associated protein 130 (SAP130) (Supplemental Figure 8F), a nuclear protein and one of the endogenous Mincle ligands (43, 44), it could significantly inhibit the release of SAP130 (Supplemental Figure 8G). These results suggest that JAML expressed in tubule cells slightly affects the activation of Mincle in macrophages, probably by influencing the release of some endogenous ligands, and participates in AKI.

### JAML deficiency also protects against AKI induced by cisplatin.

**Figure 8:**
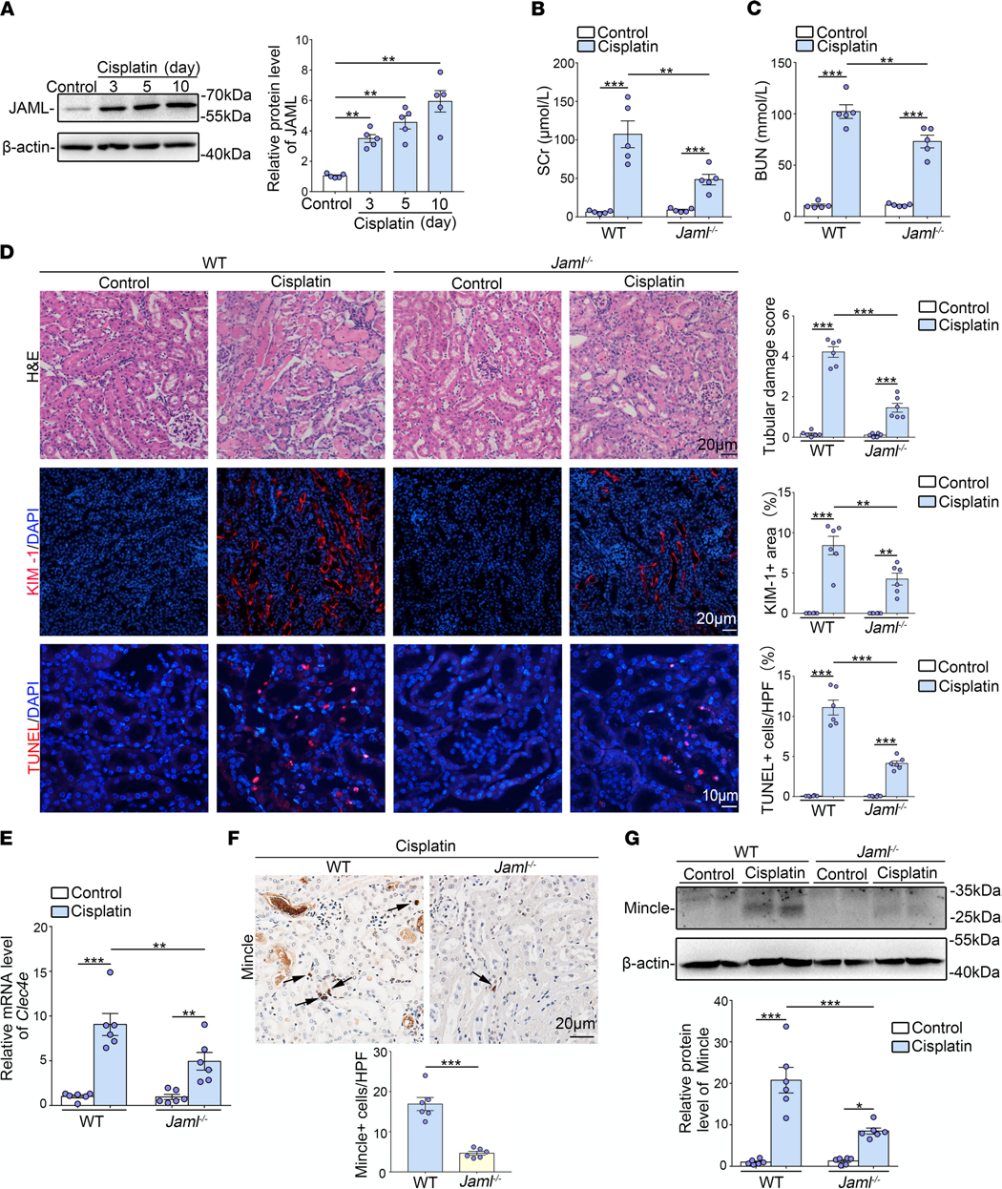
JAML deficiency protects against AKI induced by cisplatin in mice. (**A**) Representative Western blot and quantifications of JAML expression in kidneys from different groups of mice (*n* = 5). (**B** and **C**) Levels of SCr (**B**) and BUN (**C**) in different groups of mice (*n* = 5). (**D**) Representative images of H&E staining showing the morphology of kidneys and quantitative assessment of tubular damage (*n* = 6). Scale bar: 20 μm. Representative images and quantifications of immunofluorescence staining of KIM-1 (red) in kidneys from different groups of mice (*n* = 6). Scale bar: 20 μm. In situ TUNEL assays and quantification were performed to assess renal cell death (*n* = 6). Scale bar: 10 μm. (**E**) Relative mRNA levels of *Clec4e* in kidneys from different groups of mice (*n* = 6). (**F**) Representative IHC images and quantification of Mincle in kidneys from different groups of mice. Arrows indicate macrophages that express Mincle (*n* = 6). Scale bar: 20 μm. (**G**) Representative Western blot and quantifications of Mincle expression in kidneys from different groups of mice (*n* = 6). Data are mean ± SEM. *P < 0.05, **P < 0.01, ***P < 0.001. One-way ANOVA test (**A**), 2-way ANOVA test (**B**–**G**). See complete unedited blots in the supplemental material.

To confirm the broad implications of JAML signaling in the kidney, we sought to investigate whether JAML also plays a detrimental role in mice with AKI induced by cisplatin. It was found that JAML expression was also markedly increased in the kidneys from mice after cisplatin injection (Figure 8A). Compared with controls, JAML deficiency ameliorated renal dysfunction (Figure 8, B and C), tubular injury, and cell death (Figure 8D). Consistently, the levels of Mincle were also increased in the kidneys of mice with cisplatin treatment. JAML deficiency significantly decreased Mincle expression (Figure 8, E–G). Taken together, these results indicate that upregulation of JAML may be a common response in the kidney after AKI.

## Discussion

Macrophages are undoubtedly attractive therapeutic targets for AKI, with their heterogeneity and plasticity providing both opportunities and challenges. The differentiation of macrophages into morphologically and functionally distinct phenotypes M1/M2 and their clearance of dead cells (efferocytosis) have been increasingly linked to renal inflammation and repair in AKI. Therefore, achieving the full therapeutic potential of macrophages for patients with AKI requires a better understanding of the regulation of macrophage dynamics and functions. In this study, we found that JAML was significantly upregulated in kidneys from 2 separate murine AKI models including renal IRI (Figure 1) and cisplatin-induced AKI (Figure 8). *Jaml* deficiency markedly ameliorated renal dysfunction, histological lesions, and inflammatory responses in mice with AKI. Consistently, a significant increase in JAML was also observed in human kidney sections from patients with ATN, indicating that JAML may be a potential marker for AKI (Figure 1). More importantly, we demonstrated that JAML promoted AKI mainly through a macrophage-dependent mechanism (Figures 2 and 3). By generation of BM chimeric mice and tubular cell–specific *Jaml*-knockout mice, we found that JAML in BM-derived immune cells made more significant contributions to renal IRI (Figure 2), whereas JAML in renal parenchymal cells had only a slight influence on renal injury (Figure 4). Considering that macrophages and neutrophils are predominant myeloid cell types that play a critical role in renal inflammation and repair following AKI (5, 6), we further characterized the expression of JAML on renal macrophages and neutrophils individually. It was found that JAML was more significantly upregulated on infiltrating macrophages from the injured kidney compared with resident macrophages (Figure 3). However, no appreciable changes were observed on infiltrating neutrophils. Moreover, by generating macrophage-specific *Jaml*-knockout mice, which currently have a relatively high gene depletion efficiency in mature macrophages, we provided direct evidence to confirm that JAML in macrophages was the main contributor to AKI (Figure 3).

In addition to our recent finding that JAML mediates podocyte lipid metabolism in diabetic kidney disease (29), we further found that JAML regulated macrophage phenotype polarization in this study. Phenotypically polarized macrophages have the potential to switch their phenotype as tissue inflammation progresses and enters the resolution phase. However, the mechanisms that govern phenotypic polarization of macrophages and phenotypic switch are not well understood, although studies have reported that colony-stimulating factor-1, CC chemokine receptor 5, Krüppel-like factor 4, and Yes-associated protein may be the key regulators of the phenotypic switch (45–48). Here, we found that JAML not only maintained M1 phenotype of macrophages but also critically inhibited the phenotype switching from M1 to M2. Despite in vivo systems where it has been seldom observed that macrophage populations polarize to the extent observed in vitro, to make our in vitro data more solid, we still tried to evaluate macrophages’ polarization in vivo in a way that was generally acceptable as previous studies described (39, 40). Consistently, JAML deficiency promoted phenotypic polarization toward M2 after renal IRI, which was more apparent in infiltrating macrophages than in resident macrophages. Combined with the expression of JAML in 2 subtypes of macrophages after AKI, it is suggested that JAML in infiltrating macrophages may play a more important role in AKI than that in resident macrophages. However, given that JAML in resident macrophages was also increased after AKI and has a certain regulatory effect on macrophage polarization, our current study could not exclude the role of resident macrophages’ JAML in AKI. Importantly, we also found that JAML deficiency promoted macrophage efferocytosis, which is a process for the clearance of dying cells by professional and nonprofessional phagocytes and is closely linked to macrophage heterogeneity, tissue homeostasis, and M2 polarization. Efferocytosis not only is a major function of M2 macrophages but also can reinforce signaling pathways that reprogram macrophages toward an antiinflammatory phenotype in a feed-forward fashion (18, 19). Accordingly, defective efferocytosis underlies a growing list of chronic inflammatory diseases (20, 21). Therefore, further understanding the aspects of JAML-mediated efferocytosis will shed light on the key pathophysiological processes in AKI and provide novel therapeutic strategies for diseases driven by defective efferocytosis and impaired inflammation resolution.

Mechanistically, JAML deficiency affected macrophage phenotype switching, phenotype polarization, and efferocytosis, which was, at least in part, associated with a CLR Mincle-dependent mechanism. Mincle, encoded by *Clec4e*, is a member of the CLR family and is involved in the initiation of innate immune response. Moreover, as a sensor of cell death, Mincle can also recognize damage-associated molecular patterns, which induct inflammatory responses, and enable immune sensing of damaged self, which decreases dead cell clearance, thereby aggravating a vicious cycle of necroinflammation (49–51). Recent studies have indicated that Mincle is also involved in sustained inflammation after renal IRI (38). In this study, we found that JAML deficiency significantly reduced LPS- or AKI-induced Mincle expression and the activation of its direct downstream effector Syk in BMDMs (Figure 5). We further provided direct evidence showing that Mincle functions as a key regulator linking JAML to macrophage inflammation.

It should be noted that JAML expression was also induced in renal tubular cells under pathogenic conditions and tubular cell–specific JAML deletion slightly ameliorated renal IRI. Therefore, we speculated that the fate of tubular cells may be regulated by JAML. Unexpectedly, gene silencing of *Jaml* had no effect on hypoxia-induced tubular cell apoptosis. However, we found that JAML deficiency could inhibit the release of SAP130, which is one of the most important and common endogenous Mincle ligands released from dying cells under hypoxia conditions (43, 44). In fact, Mincle can recognize a series of ligands, including SAP130, cholesterol crystals, cholesterol sulfate, and β-glucosylceramide (52, 53), and has the capability of sensing endogenous and exogenous targets (51, 54). In terms of endogenous Mincle ligands, previous studies have shown that Mincle expressed on infiltrating macrophages senses dying renal tubular cells expressing β-glucosylceramide and inhibits macrophages’ phagocytic activity to induce sustained inflammation in AKI (51). In this study, we reported that JAML expressed in tubule cells can slightly affect the activation of Mincle in macrophages, probably by influencing the release of the endogenous ligands and thereby participating in the process of AKI.

**Figure 9:**
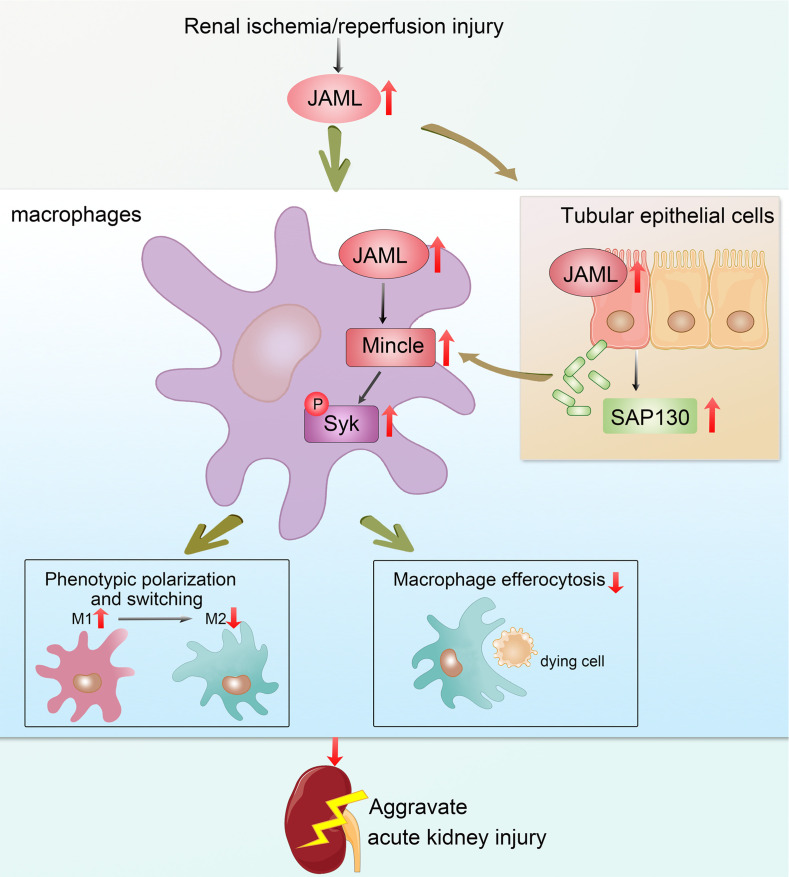
Schematic depicting that JAML promotes AKI mainly through regulating macrophage polarization and efferocytosis via C-type lectin Mincle.

We explore the potentially novel biological functions of JAML in promoting AKI by regulating macrophage polarization and efferocytosis via Mincle (Figure 9) and strengthen the concept that targeting macrophages may represent a novel therapeutic strategy for patients with AKI. Meanwhile, whether targeting JAML or macrophage therapy is sufficient to prevent renal damage in patients with AKI remains to be further established.

## Methods

### Human renal biopsy samples.

Renal biopsies had been performed as part of routine clinical diagnostic investigation, and the samples were obtained from the Department of Pathology, Shandong University School of Basic Medical Sciences. We collected the human renal biopsy samples from patients with biopsy-proven ATN as described in Supplemental Table 1. None of these patients had started dialysis therapy at the time of kidney biopsy. Normal control samples were obtained from healthy kidney poles of individuals who underwent tumor nephrectomies or renal cystectomy without having other kidney diseases.

### Mouse studies.

WT (C57BL/6J) male mice were purchased from Vital River Laboratory Animal Technology Co., Ltd. Different groups were allocated in a randomized manner, and investigators were unaware of the allocation of different groups when doing surgeries. All mice (3–5 mice per cage) were housed under standard laboratory conditions in the specific pathogen–free experimental animal center of Shandong University. Male mice (27 ± 3 g; age 8–10 weeks) were used in this study. The number of mice used for the experiments is indicated in the corresponding figure legends. All our experimental animals were kept under barrier conditions under constant veterinary supervision and did not display signs of distress or pathological changes that warranted veterinary intervention.

### Generation of global Jaml-knockout mice.

*Jaml^fl/+^* mice (C57BL/6J;129) were generated by standard homologous recombination in Shanghai Southern Model Biotechnology Development Co., Ltd. In these mice, exon 4 of *Jaml* was flanked by *loxP* sequences. Global *Jaml*-knockout mice (*Jaml*^–/–^) were obtained as described in our previous studies (29). Floxed *Jaml* mice with C57BL/6J;129 background were backcrossed with C57BL/6J mice more than 12 generations to produce congenic strains. Then, *Jaml^fl/+^* mice were crossed with EIIa-Cre transgenic mice (C57BL/6J, The Jackson Laboratory, stock no. 003724), in which the adenovirus EIIa promoter directs the expression of Cre enzyme in early mouse embryos (2- to 8-cell stage) to achieve homologous recombination between *loxP* sites, thereby triggering the deletion of exon 4 in all cells of the developing animal, including the germ cells that transmit the genetic alteration to progeny. The first generation of EIIa-Cre *Jaml^fl/+^* mice might be chimeric due to the mosaic activity of Cre recombinase. Therefore, chimeric offspring were backcrossed with C57BL/6J to generate *Jaml^+/–^* mice, which were then intercrossed for the production of *Jaml*^–/–^ mice. Mouse genotyping was performed using genomic DNA isolated from mouse tails by PCR (primers shown in Supplemental Table 2).

### Generation of macrophage-specific Jaml-knockout mice.

To obtain myeloid cell–specific deletion of *Jaml*, *Jaml* homozygous floxed (*Jaml^fl/fl^*, C57BL/6J background) mice were crossed with mice expressing Cre recombinase under the control of lysozyme 2 promoter (*LysM-Cre*, The Jackson Laboratory, stock no. 004781). Although *LysM* is not a specific marker for macrophages, *Lyz2* *(LysM)-Cre* mice currently have a relatively highly efficient gene depletion in mature macrophages and granulocytes isolated from the peritoneal cavity or derived from BM (55).

### Generation of tubular cell–specific Jaml-knockout mice.

*Jaml^fl/fl^* mice (C57BL/6J background) were hybridized with transgenic mice expressing Cre recombinase under the cadherin 16 promoter (*Ksp-Cre*) (The Jackson Laboratory, stock no: 012237) to generate tubular cell–specific *Jaml*-knockout mice (*Ksp-Cre^+^* *Jaml^fl/fl^*). *Jaml^fl/fl^* and *Ksp-Cre* mice were all used in a C57BL/6 background. Age-matched mice with 2 WT alleles and Cre expression were used as controls (*Ksp-Cre*^+^ *Jaml*^+/+^). Mouse genotyping was performed using genomic DNA isolated from mouse tails by PCR at 2 weeks of age (primers shown in Supplemental Table 2).

### Generation of murine BM chimeras.

Donor mice were sacrificed. Tibias and femurs were flushed with medium (RPMI 1640 from Gibco, Thermo Fisher Scientific, with 2% FBS, 10 U/mL heparin, and 1% penicillin and streptomycin) as described in our previous studies (56). The mixture was passed through a sterile 40 μm nylon cell strainer (Falcon, Corning) and collected in a 50 mL tube. Cells were centrifuged at 900*g* for 10 minutes at 4°C. The supernatant was discarded and the cell pellet was washed twice with 50 mL serum-free wash buffer (RPMI 1640 with 20 mM HEPES, 1% penicillin and streptomycin). After centrifugation at 900*g* for 10 minutes at 4°C, cells were resuspended in serum-free RPMI, and cell number was determined using a cell counter (TC20 automated cell counter, Bio-Rad). Moreover, 8-week-old recipient mice were lethally irradiated (9 Gy, once) and injected with 5 × 10^6^ BM cells (volume 0.2 mL) via the tail vein 6 hours after irradiation. Mice were kept on an antibiotic (1 g/L sulfamethazine in drinking water) for 2 weeks after irradiation and then switched to water without antibiotics. We used GFP-transgenic mice as donors to confirm efficient replacement. In this experiment, we used mice matched for age and genetic background and transplanted with appropriate BM (e.g., WT into WT) as controls.

### Ischemia/reperfusion model of AKI.

An established mouse model of renal IRI was performed as described previously (56, 57). Briefly, after mice were anesthetized with an intraperitoneal injection of pentobarbital sodium (30 mg/kg body weight), a midline abdominal incision was made, and bilateral renal pedicles were clipped for 40 minutes using microaneurysm clamps. At the end of the ischemic period, the vascular clamps were removed (reperfusion), and the kidneys were observed for 5 minutes to ensure reflow process. The incision was then closed and the animal was allowed to recover. During the ischemic period, body temperature was maintained at 36°C~37.5°C using a temperature-controlled heating system. After reperfusion at 24 hours, 48 hours, and 72 hours, blood samples and kidneys were collected for subsequent analysis.

### Cisplatin-induced model of AKI.

AKI in mice was also induced by a single intraperitoneal injection of cisplatin at a dose of 30 mg/kg (MilliporeSigma). At 3 days, 5 days, and 10 days after injection, mice were sacrificed, and kidney samples were collected for various analyses (56, 57).

### Cell culture and treatments.

BMDMs were isolated from tibias and femurs of mice in a procedure similar to that used for the extraction of BMDMs in the BM transplantation experiment. BM cells were cultured in DMEM supplemented with 10% FBS and macrophage colony-stimulating factor (20 ng/mL) under 37°C, 5% CO_2_ conditions. Rat proximal tubule epithelial cells (NRK-52E) were purchased from ATCC and cultured in DMEM containing 5% FBS and penicillin/streptomycin. Jurkat cells were provided by Stem Cell Bank (Chinese Academy of Sciences, Shanghai, China) and cultured in DMEM supplemented with 10% FBS and 100 U/mL penicillin plus 0.1 mg/mL streptomycin.

### Fluorescence multiplexed immunohistochemistry.

The sections were stained by using 4-color multiple fluorescent immunohistochemical staining kit (Absin). As previously described (58), the slides were baked at 65°C for 1 hour, deparaffinized in xylene, and rehydrated in gradient concentration of ethanol. Antigen retrieval was performed in citrate buffer (pH 6.0) using microwave heating. The slides were then incubated with primary antibodies, followed by a secondary horseradish peroxidase–conjugated polymer that induces the covalent binding of different fluorophores via tyramide signal amplification (TSA). This reaction was followed by additional antigen retrieval using citrate buffer in a microwave to remove the primary and secondary antibodies. Each section was stained in 2 sequential rounds, then counterstained with DAPI and mounted with Anti-Fade Fluorescence Mounting Medium (Abcam). The expression of proteins of interest was evaluated as follows: JAML, Absin 520 TSA Plus; CD68, Absin 570 TSA Plus. Antibodies used in this study are summarized in Supplemental Table 3. The images were obtained by an LSM880 laser scanning confocal microscope (ZEISS) system.

### Measurement of JAML level in mouse serum.

As previously described (29), by using mouse JAML ELISA Kit (Omnimabs), the level of JAML in serum collected from mice was measured according to the manufacturer’s instructions.

### Assessment of renal function, histological analysis, immunofluorescence staining, real-time RT-PCR, and Western blot.

These procedures were performed using standard techniques as described in Supplemental Methods.

### Tissue dissociation and flow cytometry analysis.

Mice were sacrificed and both kidneys were collected. Fresh kidneys were minced to small pieces and then placed in cold PBS with 1 mg/mL collagenase IV and 2 U/mL DNase I after taking from control mice or mice with IRI and shaking the tissues gently in a table concentrator with a constant temperature of 37°C for 30 minutes. The cell suspension was centrifuged at 400*g* for 5 minutes at 4°C after passing through a 100 μm strainer. Cell pellets were resuspended and washed with PBS, and then monocytes were isolated by using density gradient centrifugation with Percoll (Solarbio P8370). Neutrophils were isolated with the EasySep Mouse Neutrophil Enrichment Kit according to manufacturer’s instructions. The anti-CD16/CD32 antibody was used to block FcγRIII/II to minimize nonspecific antibody binding. Cells were then stained by fluorescently conjugated antibodies (10^6^ cells/1 μg) that are summarized in Supplemental Table 3. Two subtypes of macrophages from the injured mouse kidney were analyzed based on the F4/80 and CD11b fluorescence intensity. Infiltrating macrophages are CD45^+^CD11b^hi^F4/80^lo^. Resident macrophages are CD45^+^CD11b^lo^F4/80^hi^. Cell apoptosis was determined by propidium iodide–annexin V staining as described (59). Flow cytometry was performed with a CytoFLEX instrument (Beckman Coulter Biotechnology).

### Isolation of renal tubules.

Renal tubules were isolated by using a modified method for isolation of glomeruli as described in our previous studies (60). In this protocol, the final pellet was resuspended, and glomeruli containing Dynabeads were gathered by a magnetic particle concentrator (both from Invitrogen, Thermo Fisher Scientific) after washing. The residual containing renal tubules was also collected. The extracted glomeruli and tubules were put aside for protein analysis.

### TUNEL assay.

Cell death in the kidney after IRI was detected by TUNEL assay according to the manufacturer’s protocol (Roche Diagnostics).

### Microarray analysis.

The microarray experiments were performed by Sinotech Genomics Corporation. Microarray data sets have been deposited in the National Center for Biotechnology Information’s Gene Expression Omnibus under accession code GSE192532.

### Macrophage phenotype switching.

BMDMs were used for phenotype switching after they were isolated from WT and *Jaml*^–/–^ mice and cultured for 7 days. Isolated BMDMs were starved for 4 hours (M0) in medium without serum, then polarized to M2 with IL-4 (40 ng/mL) or M1 with LPS (100 ng/mL). Then, 8 hours later, M2 macrophages were switched to M1 with LPS (100 ng/mL), and M1 macrophages were treated with M2 stimulus IL-4 (40 ng/mL) for another 8 hours. Cells were harvested for the extraction of mRNA, and culture medium was collected for ELISA analysis 12 hours after the replacement of nonstimuli culture medium. The expression level of M1 or M2 markers was detected by real-time RT-PCR and ELISA analyses.

### Macrophage efferocytosis assay in vivo.

Neutrophils were collected by peritoneal lavage from donor mice 6 hours after they had been injected with zymosan A (Shanghaiyuanye Bio-Technology Co., Ltd). Neutrophils were isolated and then irradiated under a UV lamp (LightSources) to induce apoptosis. Apoptotic neutrophils labeled with PKH26 red fluorescent dye (PKH26 Red Fluorescent Cell Linker Kit, MilliporeSigma) were injected into WT or *Jaml*^–/–^ mice by intraperitoneal injection. A total of 45 minutes later, the mice were sacrificed and the peritoneum was lavaged. Efferocytosis was analyzed by flow cytometry and quantified as the percentage of CD45^+^CD11b^+^F4/80^+^ macrophages that had taken up 1 or more PKH26-labeled apoptotic cells.

### In vitro macrophage efferocytosis assay.

Jurkat cells were irradiated under a 254 nm UV lamp for 15 minutes to induce apoptosis, followed by incubation under normal cell culture conditions for 2–3 hours. This method routinely yields more than 85% apoptotic cells (ACs) as previously described (42). The ACs were resuspended at a concentration of 2 × 10^7^ cells/mL, then incubated for 2 minutes with PKH26 red fluorescent dye (2 μM/10^7^ cells). Isolated BMDMs were plated in dishes, and PKH26-labeled ACs were incubated with the macrophages for 45 minutes at a 1:5 macrophage/AC ratio. After 45 minutes, macrophages were washed 3 times with PBS to remove unbound ACs, and then the macrophages were fixed with 4% formaldehyde for 20 minutes. Images were taken by fluorescence microscopy (Olympus model U-LH100-3), and the percentage of BMDMs labeled with PKH26-tagged apoptotic Jurkat cells was quantified.

### siRNA-mediated gene silencing and adenovirus-mediated gene expression.

These procedures are provided in Supplemental Methods.

### Data availability statement.

Microarray data have been deposited in Gene Expression Omnibus (GSE192532). All other study data are included in the article and/or the supplement. Additional data related to this paper may be requested from the authors.

### Statistics.

Different groups of mice were allocated in a randomized manner, and investigators were unaware of the allocation of different groups when doing surgeries and doing outcome evaluations. Exclusion criteria prior to the start of any of the in vivo studies were death, injury requiring euthanasia, or weight loss of more than 15%. Data are expressed as mean ± SEM. Statistical analyses were performed using GraphPad Prism 8.0. Normality assumption of the data distribution was assessed using Kolmogorov-Smirnov test. Comparisons between 2 groups were performed using 2-tailed Student’s unpaired *t* test for normally distributed data and Mann-Whitney rank sum test for non-normally distributed data. Differences between multiple groups with 1 variable were determined using 1-way ANOVA followed by post hoc Tukey’s test. To compare multiple groups with more than 1 variable, 2-way ANOVA followed by post hoc Tukey’s test was used. For data with a non-Gaussian distribution, we performed a nonparametric statistical analysis using the Kruskal-Wallis test followed by Dunn’s post hoc test for multiple comparisons. Spearman’s test was implemented for statistical analyses of the correlation between 2 variables. Statistical significance was defined as *P < 0.05, **P < 0.01, ***P < 0.001, where *P* < 0.05 was the threshold for significance.

### Study approval.

All human renal biopsy samples studies were conducted in accordance with the principles of the Declaration of Helsinki and were approved by the Research Ethics Committee of Shandong University after written informed consent was obtained from the participants. All experimental protocols for animal studies were approved by the Institutional Animal Care and Use Committee of School of Basic Medical Sciences, Shandong University [Document KYLL-2017(KS)-395], and conducted in accordance with the NIH *Guide for the Care and Use of Laboratory Animals* (National Academies Press, 2011).

## Author contributions

YS and FY designed research; WH, BOW, YF, SJC, JHZ, XYZ, RKL, JCW, ZYW, MW, XJW, and ML performed research; WH, YFH, XJW, ML, WT, YZ, YSX, YS, and FY analyzed data; and YS and FY wrote the paper.

## Supplementary Material

Supplemental data

## Version 1 06/16/2022

In-Press Preview

## Version 2 07/22/2022

Electronic publication
