# Supplementary material for: JAML promotes acute kidney injury mainly through a macrophage-dependent mechanism
Source: JCI Insight. 2022 Jun 16;7(14):e158571. doi: 10.1172/jci.insight.158571 (PMC9431718; doi:10.1172/jci.insight.158571)

Supplemental Information for

JAML promotes acute kidney injury mainly through a macrophage-dependent mechanism

Wei Huang[1], Bi-Ou Wang[1], Yun-Feng Hou[2], Yi Fu[1], Si-Jia Cui[1], Jing-Han Zhu[1], Xin-Yu Zhan[1], Rong-Kun Li[1], Wei Tang[3], Ji-Chao Wu[1], Zi-Ying Wang[1], Mei Wang[1], Xiao-Jie Wang[1], Yan Zhang[1], Min Liu[1], Yu-Sheng Xie[1], Yu Sun[1*] and Fan Yi[1,4*]

* **Corresponding author**: Fan Yi or Yu Sun

Email: fanyi@sdu.edu.cn or sy5409@sdu.edu.cn

This PDF file includes:

Supplemental text
Supplemental Figures 1 to 8
Supplemental Tables 1 to 3
References

Supplemental Information Text

Materials and Methods

Assessment of renal function. High-performance liquid chromatography (HPLC) was performed to measure serum creatinine using Agilent 1100 HPLC system (Agilent Technologies, Santa Clara, CA). The procedure was performed as described previously (1, 2).

Histological analysis of renal tissues. Renal biopsy kidney tissues and the mouse kidneys were transferred to 4% paraformaldehyde (PFA) and fixed by leaving tissues at 4˚C overnight. Tissues were embedded in paraffin and cross-sectioned (4μm) for histology examination. Immunohistochemistry analysis were performed as described in our previous studies (2, 3). Isotype matched normal IgG was used as negative control to check antibody specificity. PFA-fixed kidney sections were stained with hematoxylin and eosin (H&E). The percentage of tubules in the corticomedullary junction that displayed cellular necrosis and loss of brush border were counted and scored in a blinded manner as follows: 0, none; 1, 0-10%; 2, 11-25%; 3, 26-45%; 4, 46-75%; 5, >75%. Our histological analysis was performed blinded to evaluators and in an unbiased and rigorous fashion. At least 3 sections per human subject or mice sample in 10 random microscopic vision fields at 20X, 40X or 63X magnification were selected to conduct the semi-quantitative analysis of staining by using the ImageJ software.

Immunofluorescence staining. Sections were incubated with different primary antibodies, and subsequently incubated with secondary Alexa 488 or 555 conjugated antibody (Invitrogen). Nuclei were counterstained with DAPI (Roche, Mannheim, Germany). Antibodies used in this study are summarized in Supplemental Table 3.

Real-time RT-PCR. Real-time reverse transcription PCR (RT-PCR) was performed using the UltraSYBR Mixture (CWBIO, Beijing, China). The mRNA levels for target genes were analyzed by a Bio-Rad iCycler system (Bio-Rad, Hercules, CA). The specific primers for target genes in this study are listed in Supplemental Table 2. Levels of the housekeeping gene β-actin were used as an internal control for the normalization of RNA quantity and quality differences among the samples. We calculated fold changes in gene expression normalized to β-actin by the 2-ΔΔCT method using the equation 2-2-ΔΔCT. The results were shown as fold changes compared to the control group.

Western blot analysis. Western blot analysis was performed as described previously (3). In brief, tissues as well as cell pellets from culture were resuspended in RIPA buffer containing protease inhibitor cocktail, PMSF (Phenylmethylsulfonyl fluoride) and phosphatase inhibitor. Antibodies used in this study are summarized in Supplemental Table 3. To document the loading controls, the membrane was reprobed with a primary antibody against housekeeping protein β-actin.

siRNA-mediated gene silencing. Cells were cultured in medium without antibiotics. Short interfering RNA (siRNA) for target genes or negative control siRNA were delivered into cells by the Lipofectamine 2000 reagent (Invitrogen, Carlsbad, CA) following the manufacturer's protocol. siRNA targeting sequence: 5'-CCUGGUCUCUGUAGGUUAUTT-3'; 3'-AUAACCUACAGAGACCAGGTT-5'.

Adenovirus-mediated gene expression. The adenoviruses encoding green fluorescent protein (GFP), pAdM-FH-GFP (Ad-control) and pAdM-FH-GFP-Clec4e (the Mincle overexpression construct, Ad-Mincle) with high transfection efficiency were purchased from Vigene Company (Vigene, Shandong, China). Before adenovirus infection, cells

were seeded in 6-well plates and cultured with complete culture medium. After reaching confluence, cells were incubated with culture medium containing the Ad-control and Ad-Mincle adenoviruses for 6 h, after which the medium was replaced with complete medium. After 48 h, the cells were used for gene expression analysis.

Adoptive transfer. BMDMs from wild-type (WT) or *Jaml*$^{-/-}$ donor mice were extracted and cultured in DMEM medium containing 10% BSA and M-CSF (20 ng/mL) for 7 days. The pAdM-FH-Mincle expression vector was constructed and transfected into *Jaml*$^{-/-}$ BMDMs using Lipofectamine 2000 as described previously (4). Overexpression of Mincle in macrophages from *Jaml*$^{-/-}$ mice was confirmed by Western blot analysis. Meanwhile, macrophages of recipient mouse were depleted by tail vein injection of commercially available clodronate disodium liposomes (FormuMax) (5, 6). Three days later, adoptive transfer of macrophages (MØ) (7, 8) was performed in recipient mice where macrophages were depleted, using 2×10^6 BMDM for each mouse. Three groups of chimeric mice were constructed: WT MØ → WT, *Jaml*$^{-/-}$ MØ → *Jaml*$^{-/-}$, mincle overexpressed *Jaml*$^{-/-}$ MØ →*Jaml*$^{-/-}$.

In vivo kidney efferocytosis assay. As previously described (9, 10), the mouse kidney tissue was paraffin-embedded and sectioned, followed by staining of the sections (4μm) with TUNEL reagents and an antibody to CD68. In situ efferocytosis was quantified by counting TUNEL$^+$ nuclei that were associated with CD68$^+$ macrophages (associated), indicative of efferocytosis, or not associated with macrophages (free). Macrophage-associated apoptotic cells followed the criteria of TUNEL$^+$ nuclei surrounded by or in contact with neighboring CD68$^+$ macrophages. Free apoptotic cells exhibited nuclear condensation, loss of antibody CD68 reactivity, and were not in contact with neighboring

macrophages. The images were obtained by a LSM880 laser scanning confocal microscope (ZEISS, Germany) system. The ratio of macrophage-associated apoptotic cells to free apoptotic cells was calculated.

Supplemental Figures

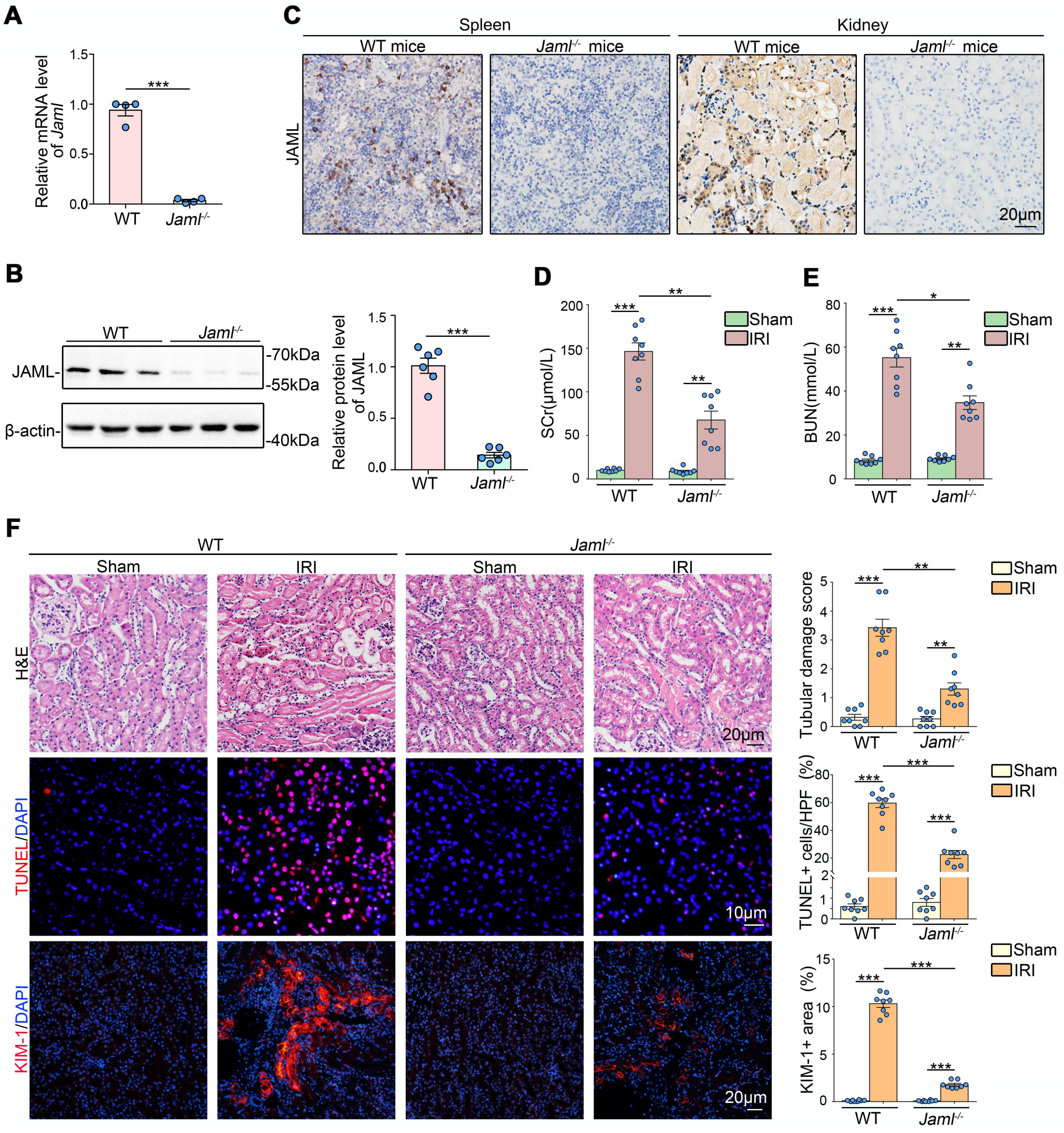

Supplemental Figure 1. JAML deficiency protected against renal injury in mice with renal IRI. **(A)** Relative mRNA level of *Jaml* in the kidney from *Jaml* $^{-/-}$ mice (*n* = 4). **(B)** Representative Western blot and quantifications of JAML expression in the kidney from *Jaml* $^{-/-}$ mice (*n* = 6). **(C)** Representative IHC images of JAML in the spleen and injured kidney from both WT and global *Jaml* knockout (*Jaml* $^{-/-}$) mice (*n* = 3). Scale bar: 20μm. **(D)** SCr concentration in different groups of mice after renal IRI (*n* = 8). **(E)** BUN levels of different groups of mice after renal IRI (*n* = 8). **(F)** Representative images of H&E staining showing the morphology of kidney and quantitative assessment of tubular damage Scale bar: 20μm. *In situ* TUNEL assays and quantification were performed to assess renal cell death. Scale bar: 10μm. Representative images and quantification of immunofluorescence (IF) staining of kidney injury molecule 1 (KIM-1) (red) in the kidney from different groups of mice (*n* = 8). Scale bar: 20μm. HPF, high power field. Data are mean ± SEM. **P*<0.05, ***P*<0.01, ****P*<0.001. Two-tailed Student's unpaired t test (A and B), Two-way ANOVA test (D-F).

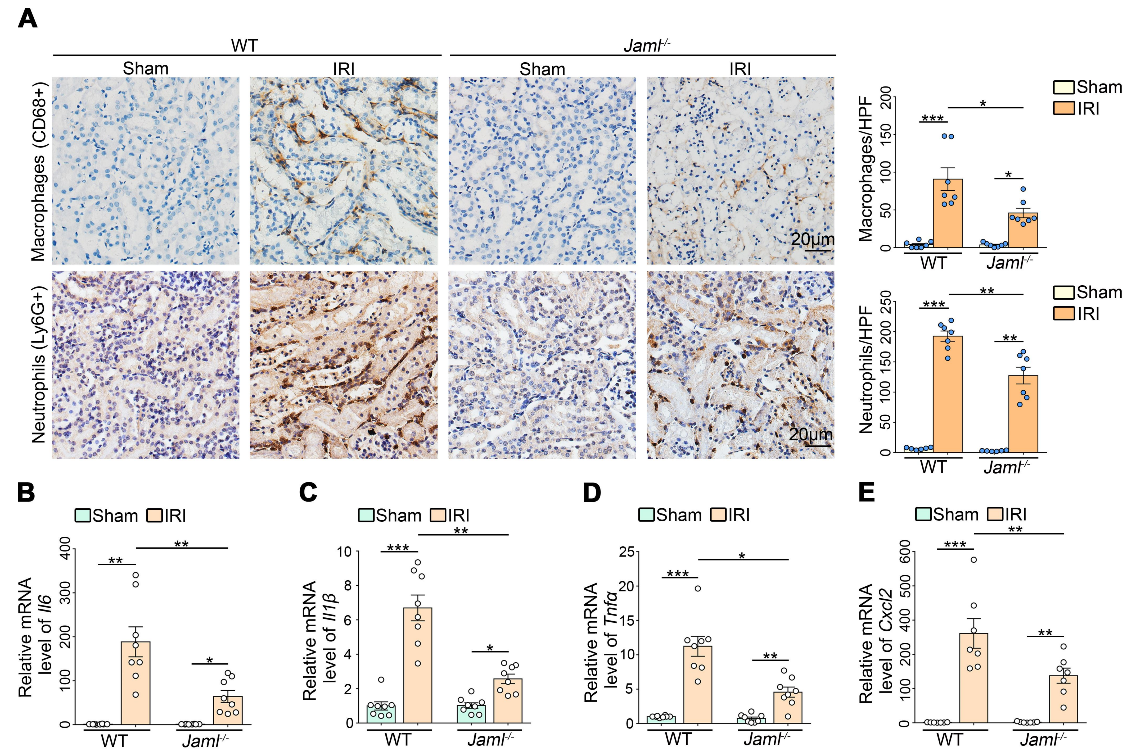

Supplemental Figure 2. JAML deficiency alleviated inflammatory responses in mice with renal IRI. (A) Representative IHC images and quantifications of macrophages (CD68+) and neutrophils (Ly6G+) in the kidney from mice with renal IRI (*n* = 7). HFD, high-power field. Scale bar: 20μm. **(B-E)** Relative mRNA levels of proinflammatory mediators including interleukin-6 (*Il6*) (*n* = 8) (B), *Il1β* (*n* = 8) (C) , tumor necrosis factor α (*Tnfα*) (*n* = 8) (D) and C-X-C motif chemokine ligand 2 (*Cxcl2*) (*n* = 7) (E) in the kidney from different groups of mice. Scale bar: 20μm. HPF, high power field. Data are mean ± SEM. **P*<0.05, ***P*<0.01, ****P*<0.001.Two-way ANOVA test (A-E).

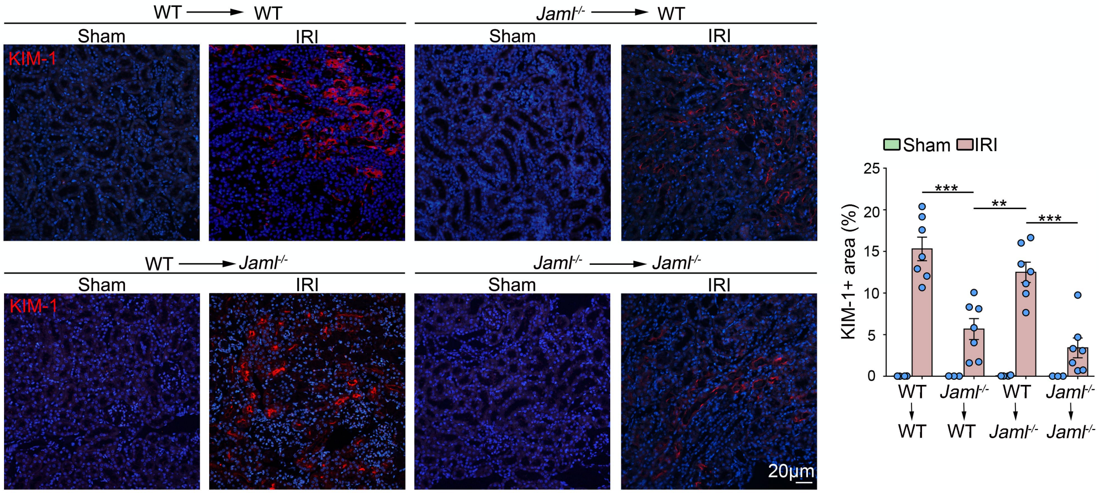

Supplemental Figure 3. BM-derived immune cells with JAML deficiency apparently alleviated renal damage after IRI. Representative images and quantifications of IF staining of KIM-1 (red) in the kidney from different groups of mice after BM transplatation (n = 7), Scale bar: 20μm. Data are mean ± SEM. *P<0.05, **P<0.01, ***P<0.001. Two-way ANOVA test analysis.

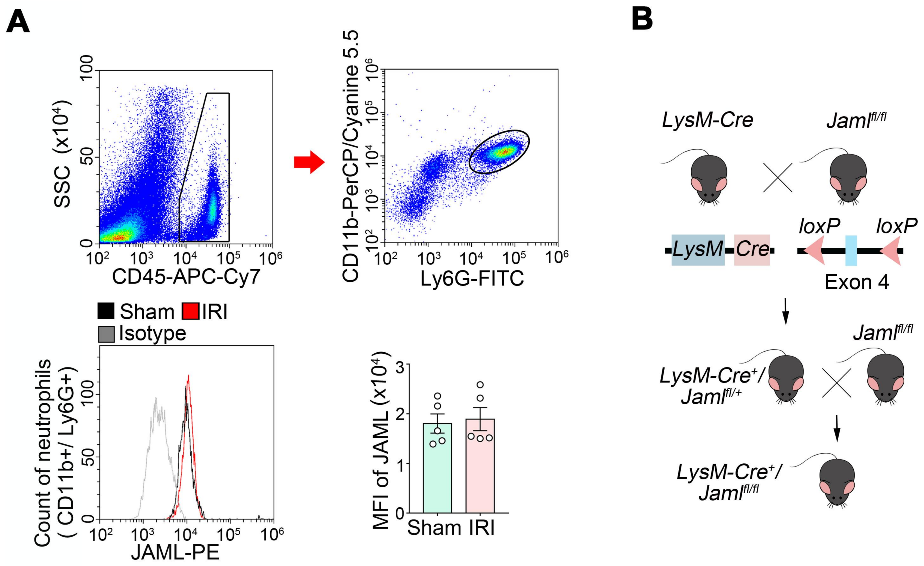

Supplemental Figure 4. Cell-surface JAML expression on neutrophils from kidney in mice with IRI and generation of macrophage-specific *Jaml* knockout mice. (A) Representative histogram showing cell-surface JAML expression of neutrophils (n = 5) and quantitative analysis of the mean fluorescence intensity (MFI) of JAML-PE. PE, phycoerythrin. **(B)** Experimental scheme for generating of conditional knockout mice in which *Jaml* is specifically deleted in myeloid cells (*LysM-Cre*$^{+}$/ *Jaml*$^{fl/fl}$) by using *Cre–LoxP* recombination system. Exon 4 is deleted upon *LysM-Cre* mediated recombination. Two-tailed Student's unpaired t test (A).

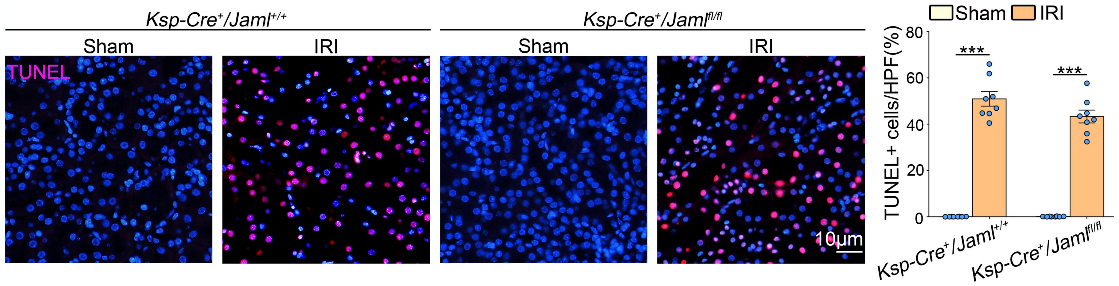

Supplemental Figure 5. JAML in renal tubular cells has a slight influence on renal injury. In situ TUNEL assays and quantification were performed to assess renal cell death in the kidney from different groups of mice (*n* = 7). Nuclei were revealed using DAPI staining (blue). Scale bar: 10μm. Data are mean ± SEM. **P*<0.05, ***P*<0.01, ****P*<0.001. Two-way ANOVA test analysis.

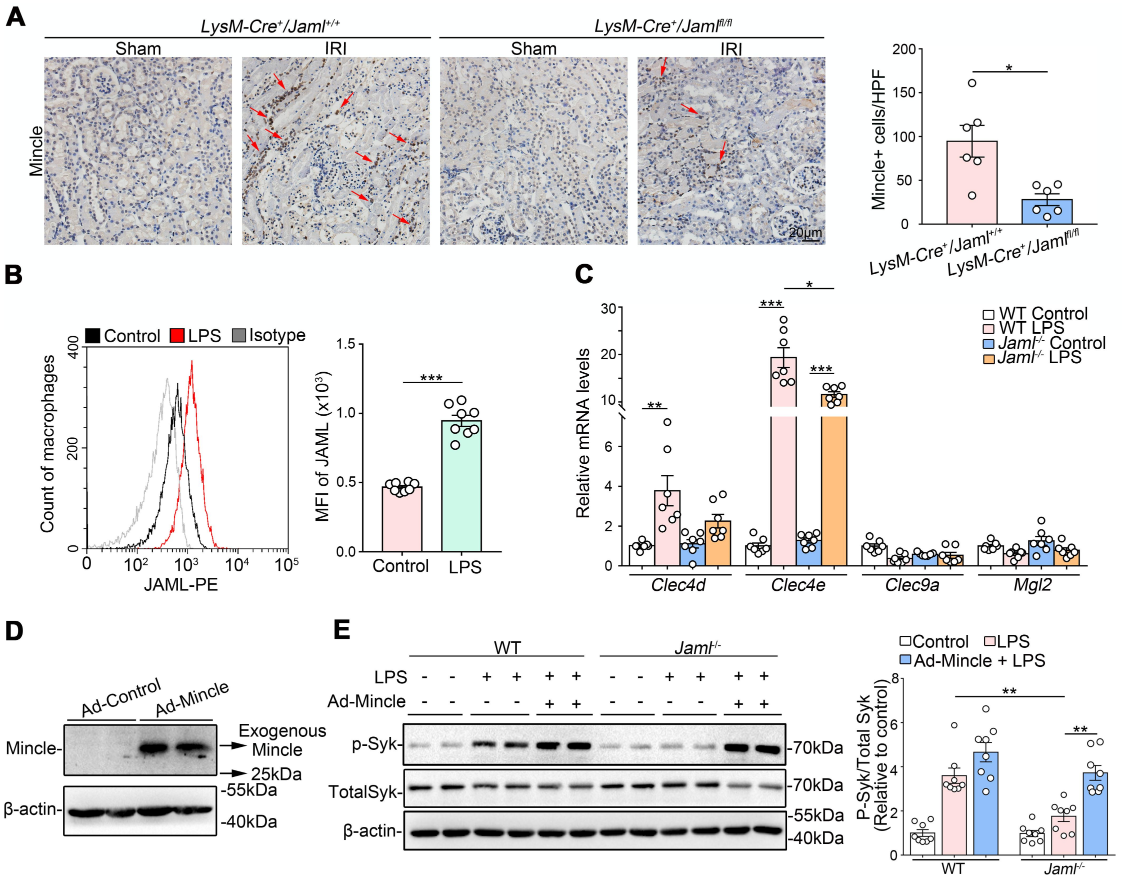

Supplemental Figure 6. Up-regulation of JAML facilitated C-type lectin receptor Mincle signaling in macrophages. (A) Representative IHC images and quantification of Mincle in different groups of mice (*n* = 6). **(B)** Representative flow cytometry histogram showing cell-surface JAML expression on LPS-treated BMDMs and quantitative analysis of MFI of JAML-PE (*n* = 8). **(C)** Relative mRNA levels of C-type lectin members including *Clec4d, Clec4e* (Mincle), *Clec9a and Mgl2* in different groups of BMDMs (*n* = 7). **(D)** Representative Western blot showing the overexpression of Mincle in BMDMs by using a recombinant adenovirus vector (Ad-Mincle) (*n* = 4). **(E)** Representative Western blot and quantifications of phosphorylated and total Syk in different groups of BMDMs (*n* = 8). Data are mean ± SEM. **P*<0.05, ***P*<0.01, ***P<0.001. Two-tailed Student's unpaired t test (A and B), Two-way ANOVA test analysis (C and E).

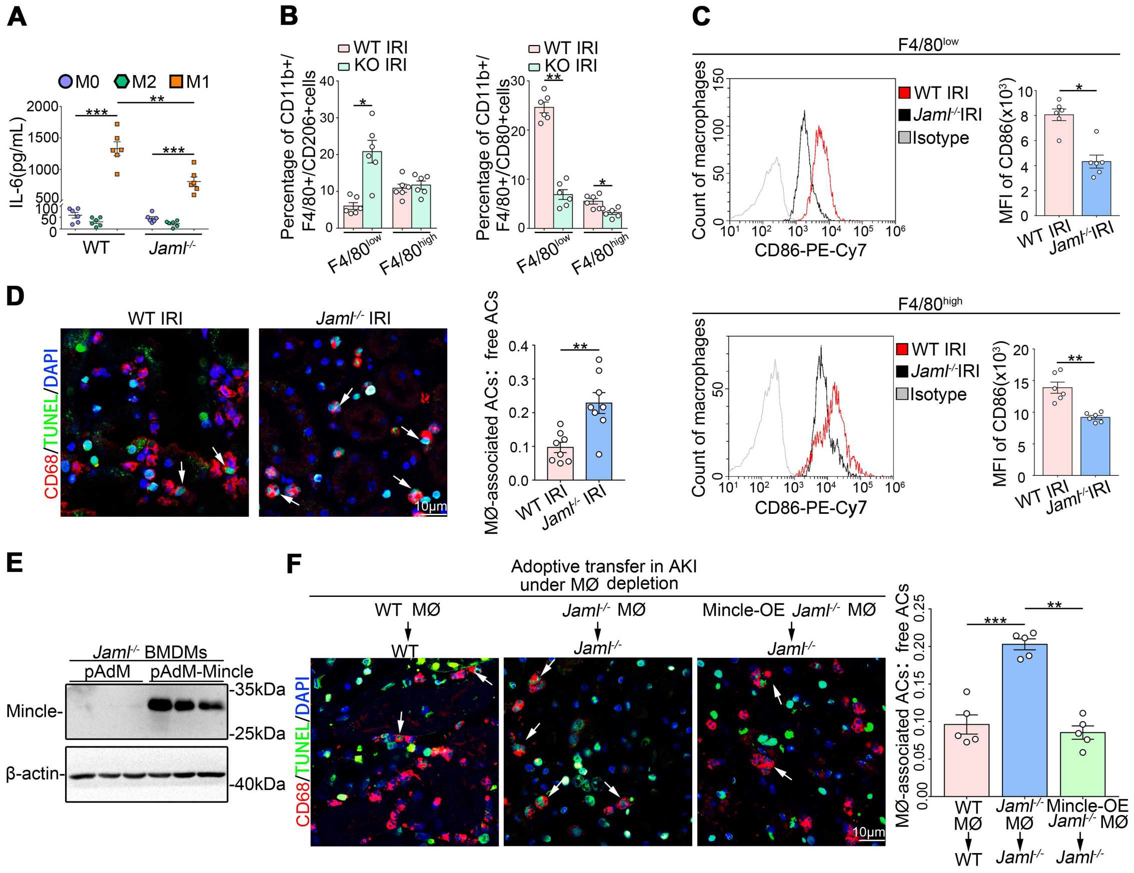

Supplemental Figure 7. JAML regulated macrophage phenotypic polarization and efferocytosis via a Mincle-dependent mechanism. (A) The level of IL-6 in supernatant from BMDMs with different treatments was measured by ELISA (*n* = 6). **(B)** The histogram bars show the percentage intensity change in each cell population (M2 and M1) in *Jaml* $^{-/-}$ mice after IRI compared to WT mice (n = 6). **(C)** Representative flow cytometry histogram showing cell-surface marker CD86 (M1) expression on two subtypes of macrophages and quantitative analysis of MFI of CD86-PE-Cy7 (*n* = 6). **(D)** Representative IF staining of CD68 (red) and TUNEL (green) in sections from injured kidney of WT and *Jaml* $^{-/-}$ mice. Nuclei were revealed using DAPI staining (blue). The percentage of macrophage-associated TUNEL+ apoptotic cells to free apoptotic cells was quantified (n = 8). **(E)** Representative Western blot showing the overexpression of Mincle in *Jaml*$^{-/-}$ BMDMs by liposomes mediated transfection (*n* = 5). MØ, macrophages. **(F)** Representative IF staining of CD68 (red) and TUNEL (green) in sections from injured kidney of different groups of mice with or without macrophage deletion or transfer. The percentage of macrophage-associated TUNEL+ apoptotic cells to free apoptotic cells was quantified (n = 5). Data are mean ± SEM. **P*<0.05, ***P*<0.01, ****P*<0.001. Two-way ANOVA test analysis (A, B, F). Two-tailed Student's unpaired t test (C, D).

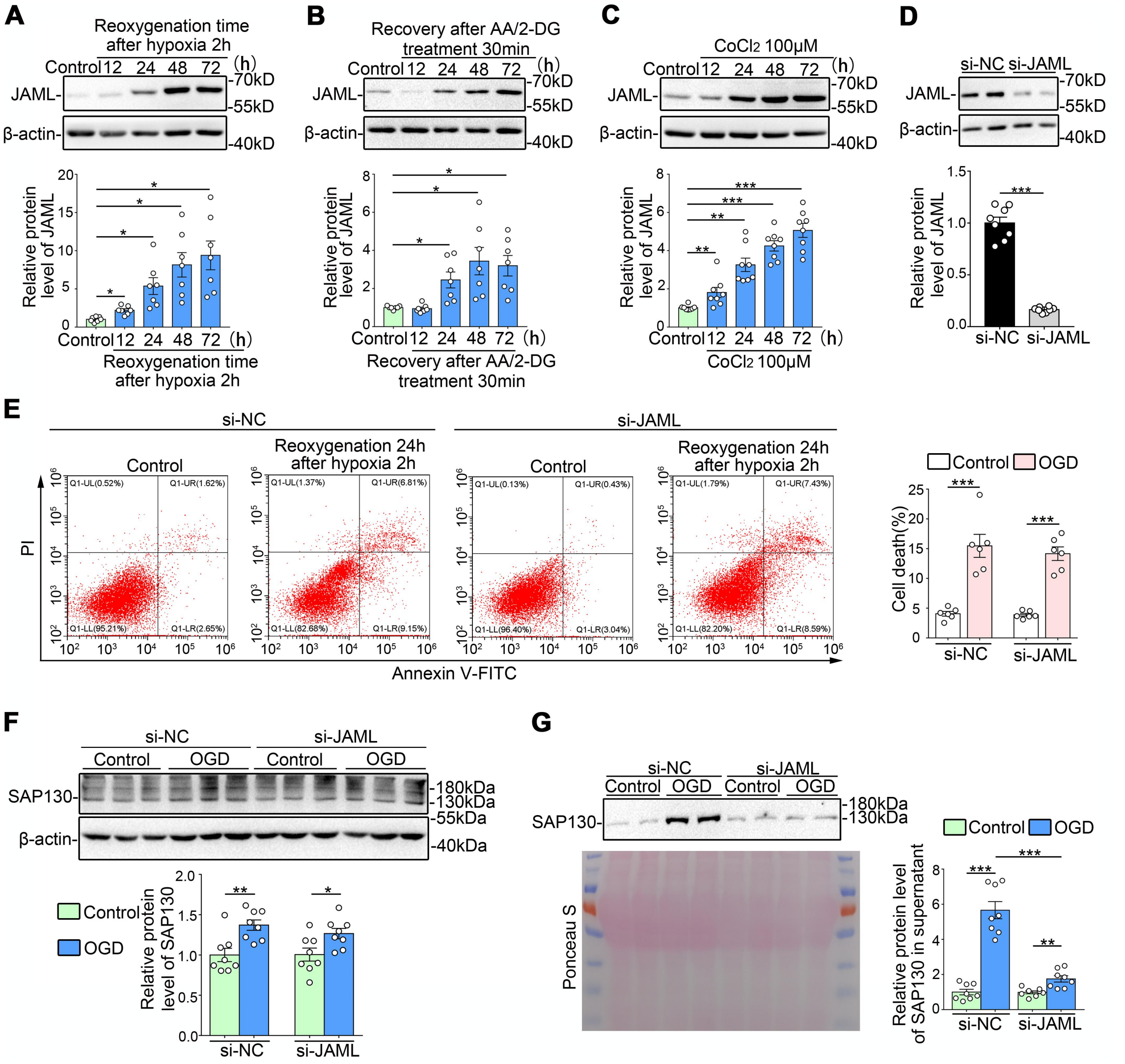

Supplemental Figure 8. Gene silencing of JAML inhibited the release of endogenous Mincle ligands from proximal tubule epithelial cells. (A-C) Representative Western blot and quantifications of JAML expression in NRK-52E cells under different treatments, such as OGD condition (cultured in a hypoxic environment for 2h (0.1% O2) followed by reoxygenation at different time points) (A, *n*=7), chemical anoxia/recovery condition (cultured in glucose-free medium with antimycin A (AA, 10 mM)/2-deoxyglucose (2-DG, 25 mM) for 30 min (anoxia) to inhibit aerobic and substrate-dependent adenosine triphosphate generation, and then in glucose-replete complete growth medium for different time (recovery))(B, *n* = 7) and $CoCl_2$ treatments for different times (C, *n* = 8). **(D)** Representative Western blot and quantifications of JAML in liposomes mediated control siRNA (si-NC) or JAML siRNA (si-JAML) transfected NRK-52E cells (*n* = 8). **(E)** Summarized data showing cell death determined by flow cytometric analysis in NRK-52E cells with different treatments (*n* = 6). (**F**) Representative Western blot and quantifications of SAP130 expression in NRK-52E cells with different treatments (*n* = 8). **(G)** Representative Western blot and quantifications of SAP130 in supernatant from NRK-52E cells with different treatments. The Ponceau S Staining was used as a loading control (*n* = 8). Data are mean ± SEM. **P*<0.05, ***P*<0.01, ****P*<0.001. One-way ANOVA test (A-C), Two-tailed Student's unpaired t test (D), Two-way ANOVA test analysis (E-G).

Supplemental Tables

Supplemental Table 1. Clinical characteristics in the normal human control subjects or subjects with acute tubular necrosis.

Number	Age (year)	Sex	SCr (μmol/L)	BUN (mmol/L)	ACR (mg/24h)	α1-MG (mg/L)	Complications
Normal human control subjects							
1	25	M	74	5.2	NA	NA	SRC
2	34	M	99	5.7	NA	NA	RN
3	47	M	83	7.2	NA	NA	RN
4	58	F	60	3.4	NA	NA	hematuresis
5	36	M	63	5	NA	NA	RN
6	65	M	122	7.2	NA	NA	RN
7	60	M	NA	NA	NA	NA	RN
Subjects with acute tubular necrosis							
Number	Age (year)	Sex	SCr (μmol/L)	BUN (mmol/L)	ACR (mg/24h)	α1-MG (mg/L)	Complications
1	53	F	159	NA	NA	NA	AKT
2	26	F	143	NA	0.71	20.2	nephritis
3	57	M	183	10.8	NA	NA	ARF/NS
4	44	F	186	NA	NA	NA	AKI/NS
5	38	M	183	NA	9.69	NA	NS
6	13	M	213	10.3	NA	88.6	ARI
7	47	M	113	11.6	NA	NA	hypertension
8	19	M	75	NA	2.96	NA	NS
9	53	M	170	NA	NA	NA	NS
10	25	F	145	NA	2.66	NA	AKI
11	34	M	183	13.53	NA	23.4	CKD III
12	22	F	110	8.4	NA	NA	nephritis
13	21	F	90	NA	2.86	NA	nephritis
14	8	M	256	NA	NA	NA	ARI
15	36	M	126	7	NA	NA	hypertension
16	45	M	252	NA	NA	NA	NS
17	30	M	173	NA	1.968	NA	NS
18	15	M	216	NA	NA	NA	IN
19	45	F	61	NA	2.79	NA	NS
20	56	M	100	NA	NA	NA	NS
21	43	M	74	NA	NA	NA	NS
22	37	F	194	10.2	NA	NA	NS
23	51	F	127	NA	0.25	NA	AKI
24	21	M	137	NA	NA	NA	NS
25	38	F	128	NA	0.47	NA	RI
26	33	M	107	NA	NA	51.6	NS
27	52	M	184	11.05	NA	NA	AKI
28	67	M	99	7.7	NA	NA	NS
29	33	F	127	NA	NA	NA	nephritis
30	55	M	94	NA	4.48	NA	NS
31	14	M	99	NA	NA	NA	AKI
32	57	M	194	18.1	NA	NA	nephritis

33	48	F	130	16.4	NA	NA	AKI
34	31	M	212	NA	NA	NA	nephritis
35	58	F	140	NA	NA	NA	CRI
36	21	M	137	NA	2.3	NA	NS
37	67	M	94	NA	NA	NA	nephritis
38	68	M	137	10.1	1.29	NA	NS
39	32	F	127	NA	1.86	NA	NS
40	72	M	147	NA	NA	NA	nephritis
41	33	M	142	NA	NA	NA	nephritis
42	69	F	127	18.3	NA	NA	T2DM
43	31	M	139	NA	NA	NA	nephritis
44	12	F	66.9	22.1	NA	NA	nephritis

ACR, albumin creatinine ratio; AKI, acute kidney injury; AKT, allogeneic kidney transplantation; α1-MG, α-1 microglobulin; ARF, acute renal failure; ARI, acute renal insufficiency; BUN, blood urea nitrogen; CKD III, chronic kidney disease stage 3; CRI, Chronic renal insufficiency; F, female; IN, Interstitial nephritis; M, male; NA, not available; NS, nephrotic syndrome; RI, Renal impairment; RN, renal neoplasms; SCr, creatinine; SRC, simple renal cyst; T2DM, type 2 diabetes mellitus.

Supplemental Table 2. Primers for tail PCR genotyping and real-time RT-PCR.

Gene	Forward (5' to 3')	Reverse (5' to 3')
Jaml	CTGCCAGGCTTGACCGTTTC	CGCTGGACAACACATCCCAT
Tnfα	GTCCGGGCAGGTCTACTTTG	GGGGCTCTGAGGAGTAGACA
Cxcl2	CTGCCAAGGGTTGACTTCAAG A	CTTCAGGGTCAAGGCAACT
Il1β	AATGCCACCTTTTGACAGTGA TG	ATGTGCTGCTGCGAGATTTG
Il6	GCCTTCTTGGGACTGATGCT	GCCATTGCACAACTCTTTTCTCA
iNOS	CAATGGCAACATCAGGTCGG	CGTACCGGATGAGCTGTGAA
Arg1	AAGACAGCAGAGGAGGTGAA GAG	TGGGAGGAGAAGGCGTTTGC
Ccl8	GCCAGATAAGGCTCCAGTCA	GGTCTGGAAAACCACAGCTTCC
Clec4d	GACTGGCATAGCCTCACAAGA	CATAATCAGCCTCCAAACCCT
Clec4e	AGGAGCTTTCCTGCTACAGT	CCAGGTCAAGGTTGTCGTAGA
Clec1b	ACTGGATGTGGGAGGATAGC	AGCAGTTGGTCCACTCTTGTC
Clec2h	GTGAACCTTGCTACACCGCA	ATGCTACGCAGGAGGACTGA
Clec7a	GCATCCCAAACTACAGGAGGT	CGGTGAGACGATGTTTGGCT
Clec9a	TGCCAATCCCCTAGCAAATGT	CAAGCCCATACAGACCACACA
Clec12a	CGATGCTTCCCATTCACAGC	ATGCCTCCTAAGACCCCCAT
Mgl2	CTGAGAAGTCGTGGCCTGAA	CCTCCAATTCTTGAAACCTTTGT
β-actin	GGCTGTATTCCCCTCCATCG	CCAGTTGGTAACAATGCCATGT
Jaml$^{fl/fl}$	GTCATCCAGCTAGGGAACCG	GCAGGCCCTGTGGATAACCT
Global *Jaml* knockout	AGTGAGCCAGAGGGTGTGGA	GTTAAAGCGAAAGTGAGGTT
Ksp-Cre	GCAGATCTGGCTCTCCAAAG	AGGCAAATTTTGGTGTACGG
Internal positive control	CAAATGTTGCTTGTCTGGTG	GTCAGTCGAGTGCACAGTTT
LysM-Cre	CAGCATTGCTGTCACTTGGTC	ATTTGCCTGCATTACCGGTCG

Supplemental Table 3. The list of antibodies used in this study.

Antibody	Source	Fluorochrome	Provider	Catalog
JAML	Goat	--	R&D Systems	AF3449
JAML	Rabbit	--	Abcam	ab183714
CD68	Mouse	--	Abcam	ab955
CD68	Rabbit	--	Abcam	ab125212
Ly6G	Rat	--	Santa Cruz Biotechnology	sc-53515
β-actin	Rabbit	--	ZEN BIO	380624
KIM-1	Rabbit	--	BOSTER	BA3537
Mincle	Rat	--	MBL	D266-3
Mincle	Rabbit	--	Absin	abs127697
Phospho-Syk	Rabbit	--	Cell Signaling Technology	2717
Syk	Rabbit	--	Cell Signaling Technology	13198
SAP130	Rabbit	--	ZEN BIO	383077
JAML	Armenian Hamster	PE	Biolegend	128503
CD45	Rat	APC/Cyanine7	Biolegend	103116
CD45	Rat	FITC	Biolegend	103108
CD11b	Rat	FITC	Biolegend	101206
CD11b	Rat	PerCP/Cyanine5.5	Biolegend	101228
F4/80	Rat	PE	Biolegend	123110
F4/80	Rat	APC	Biolegend	123116
Ly-6G	Rat	FITC	Biolegend	127605
CD80	Armenian Hamster	PE-Cy7	Biolegend	104734
CD86	Rat	PE-Cy7	Biolegend	105014
CD206	Rat	Alexa Fluor 647	Biolegend	141712

References

1. Li Q, et al. NLRC5 deficiency protects against acute kidney injury in mice by mediating carcinoembryonic antigen-related cell adhesion molecule 1 signaling. *Kidney Int.* 2018;94(3):551-66.
2. Fang W, et al. Gpr97 Exacerbates AKI by Mediating Sema3A Signaling. *J Am Soc Nephrol.* 2018;29(5):1475-89.
3. Fu Y, et al. Elevation of JAML Promotes Diabetic Kidney Disease by Modulating Podocyte Lipid Metabolism. *Cell Metab.* 2020;32(6):1052-62 e8.
4. Vadevoo SMP, et al. The macrophage odorant receptor Olfr78 mediates the lactate-induced M2 phenotype of tumor-associated macrophages. *Proc Natl Acad Sci U S A.* 2021;118(37).
5. Shi J, et al. Cre Driver Mice Targeting Macrophages. *Methods Mol Biol.* 2018;1784:263-75.
6. Chow A, et al. Bone marrow CD169+ macrophages promote the retention of hematopoietic stem and progenitor cells in the mesenchymal stem cell niche. *J Exp Med.* 2011;208(2):261-71.
7. Lv LL, et al. The pattern recognition receptor, Mincle, is essential for maintaining the M1 macrophage phenotype in acute renal inflammation. *Kidney Int.* 2017;91(3):587-602.
8. He H, et al. Perivascular Macrophages Limit Permeability. *Arterioscler Thromb Vasc Biol.* 2016;36(11):2203-12.
9. Yurdagul A, et al. Macrophage Metabolism of Apoptotic Cell-Derived Arginine Promotes Continual Efferocytosis and Resolution of Injury. *Cell Metab.* 2020;31(3):518-33 e10.
10. Cai W, et al. STAT6/Arg1 promotes microglia/macrophage efferocytosis and inflammation resolution in stroke mice. *JCI Insight.* 2019;4(20).

Full unedited gels for representative western blot and RT-PCR agarose gels. Related to Figure 1-8 and Supplemental Figure 1-8.

Full unedited gel for Figure 1D

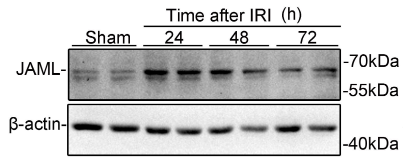

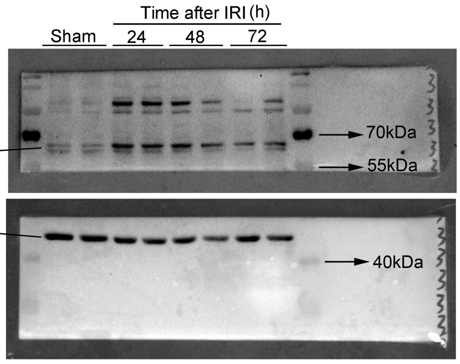

Full unedited gel for Figure 3B

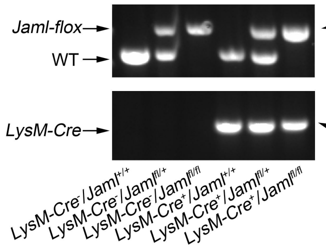

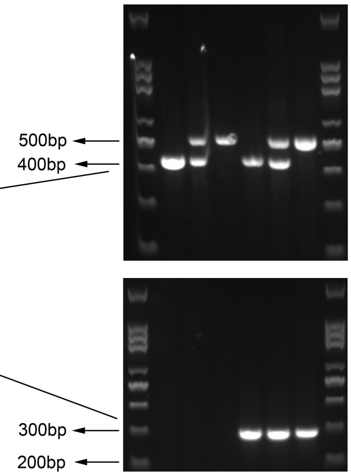

Full unedited gel for Figure 3C

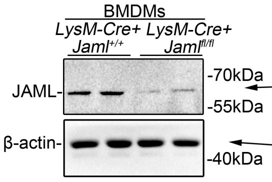

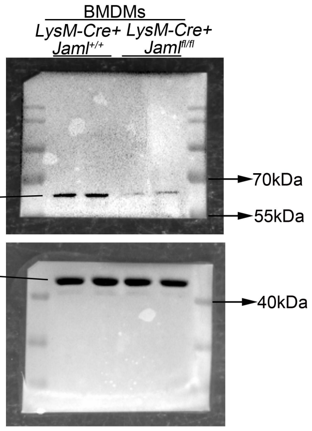

Full unedited gel for Figure 4B

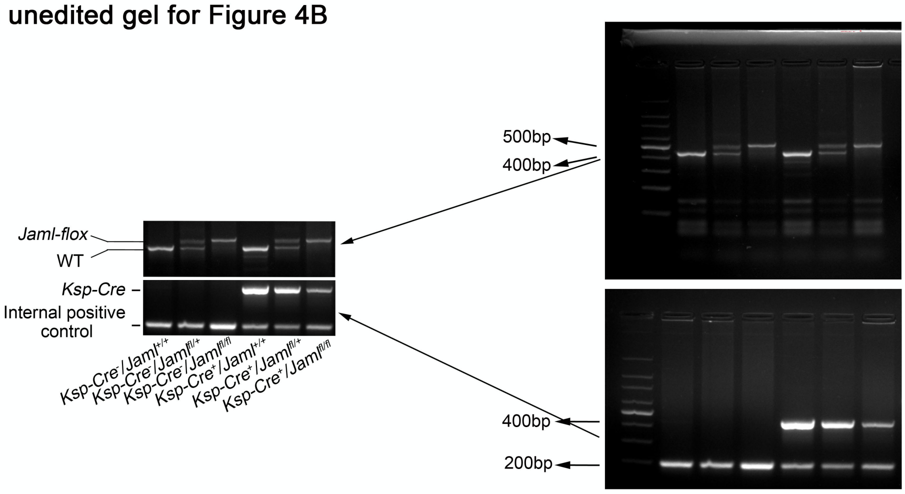

Full unedited gel for Figure 4C

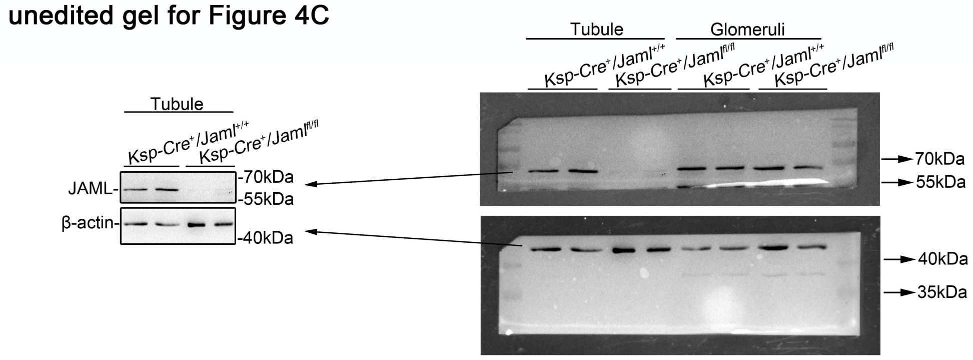

Full unedited gel for Figure 5C

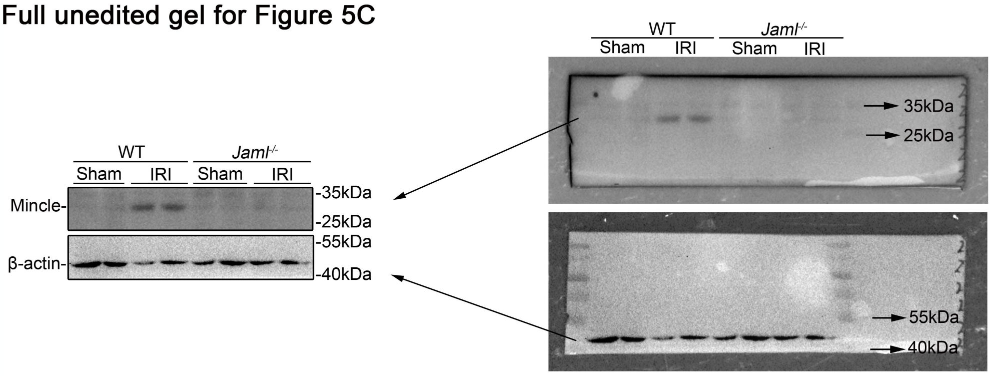

Full unedited gel for Figure 5E

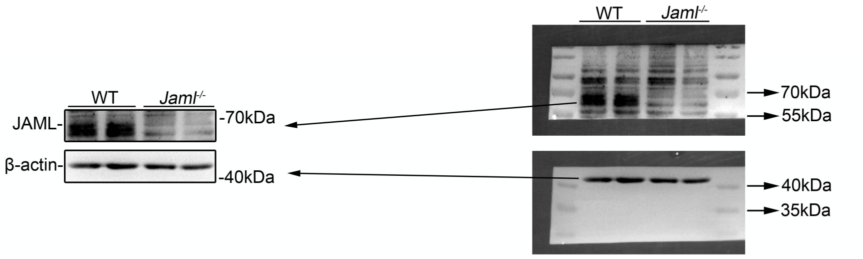

Full unedited gel for Figure 5J

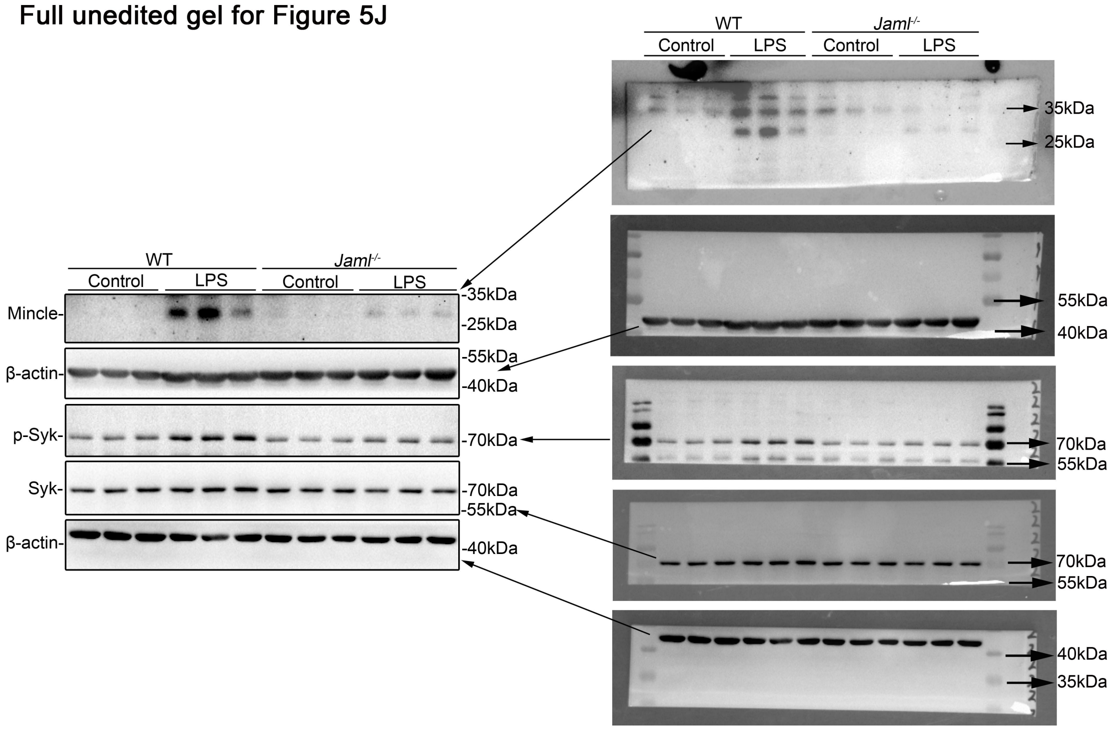

Full unedited gel for Figure 8A

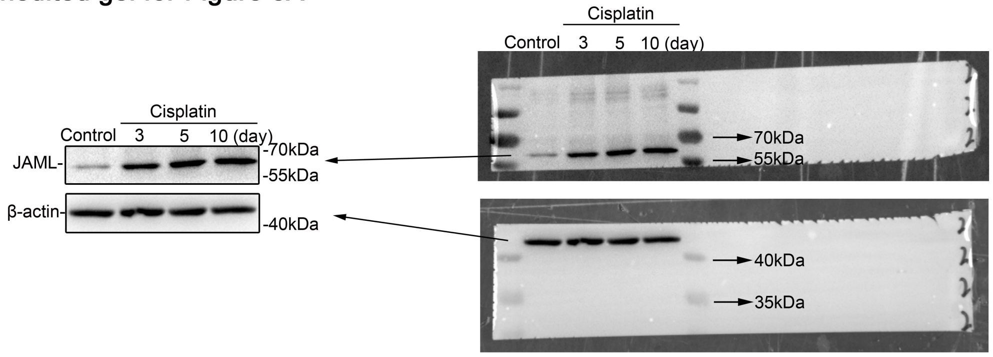

Full unedited gel for Figure 8G

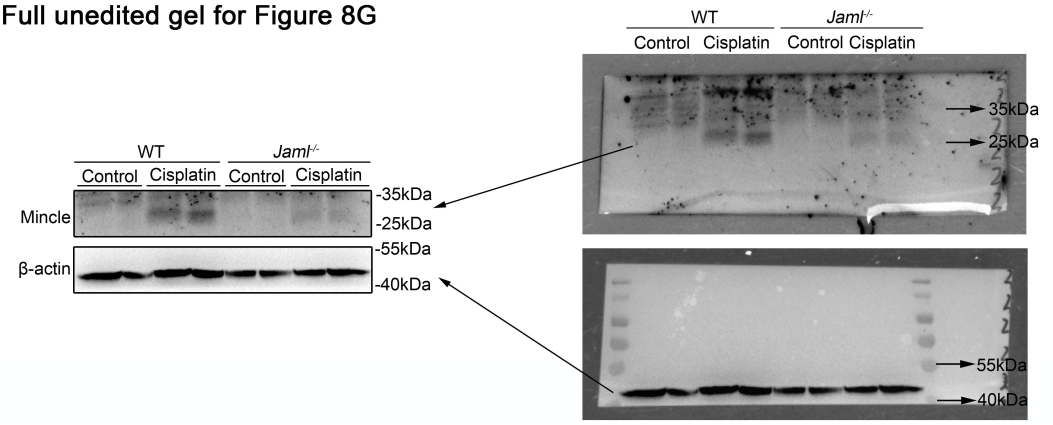

Full unedited gel for Supplemental Figure 1B

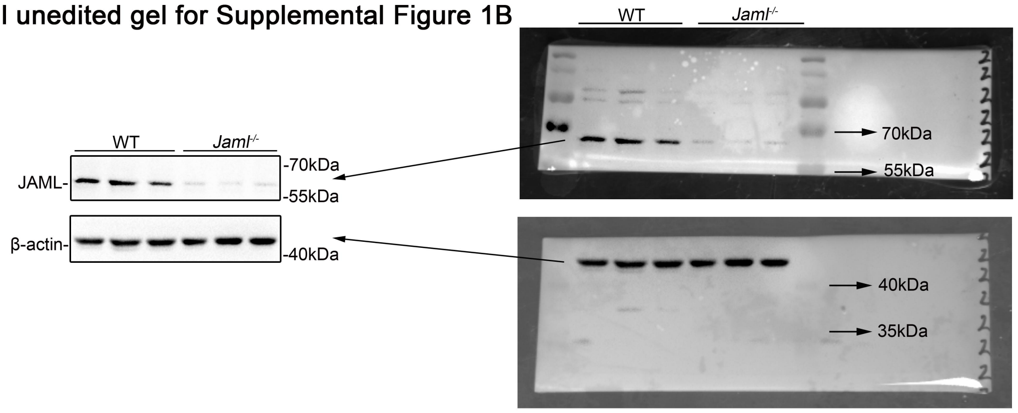

Full unedited gel for Spplemental Figure 6D

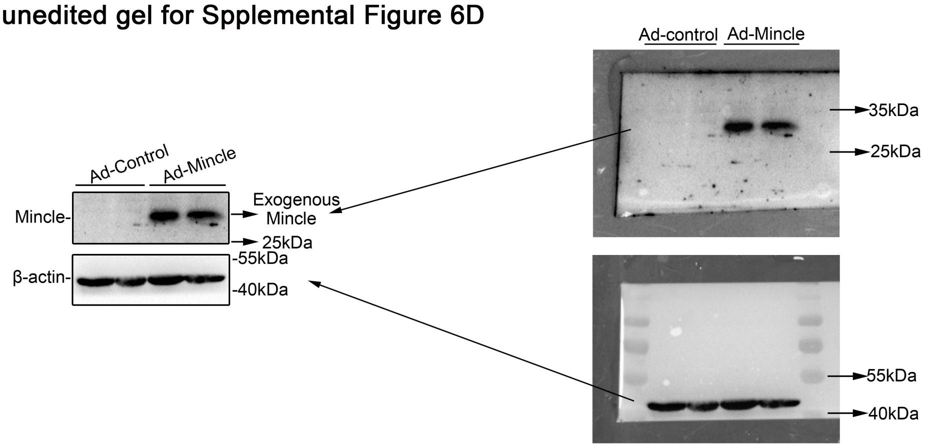

Full unedited gel for Supplemental Figure 6E

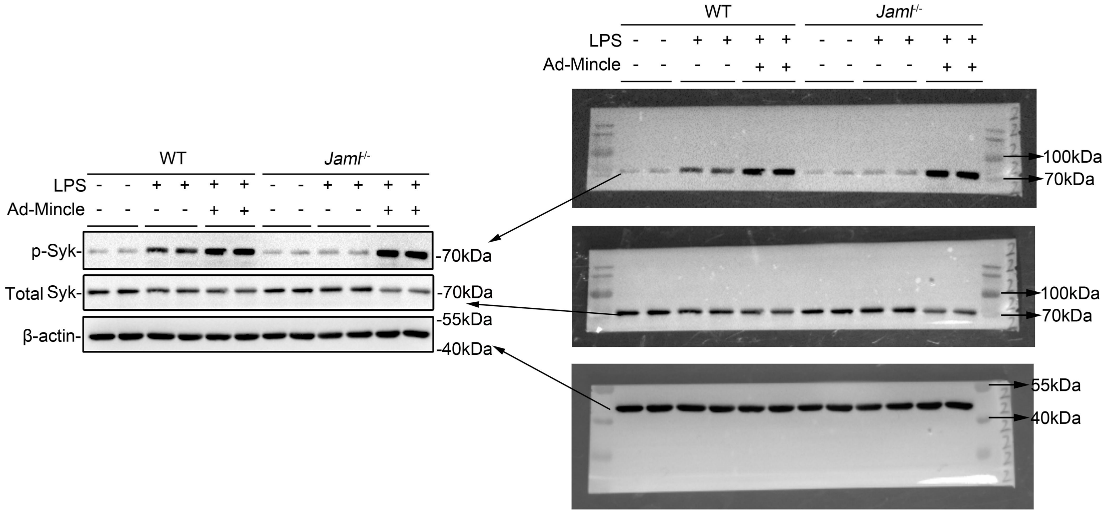

Full unedited gel for Supplemental Figure 7E

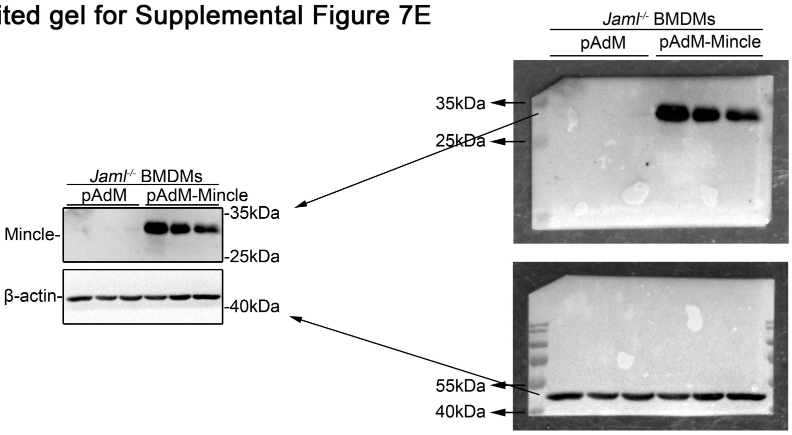

Full unedited gel for Supplemental Figure 8A

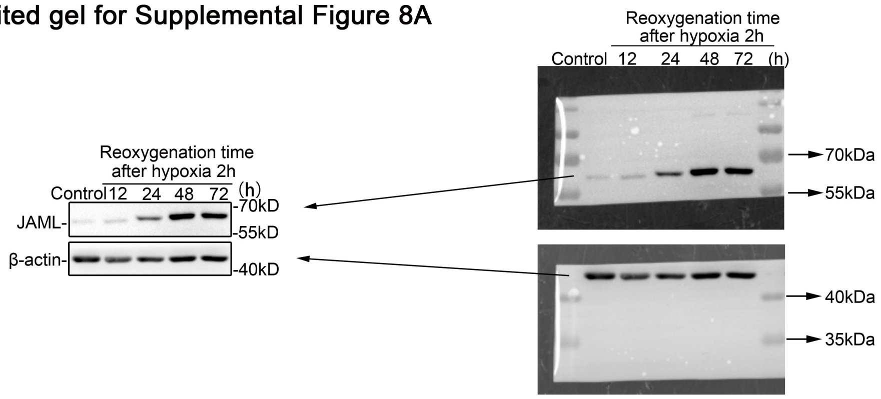

Full unedited gel for Supplemental Figure 8B

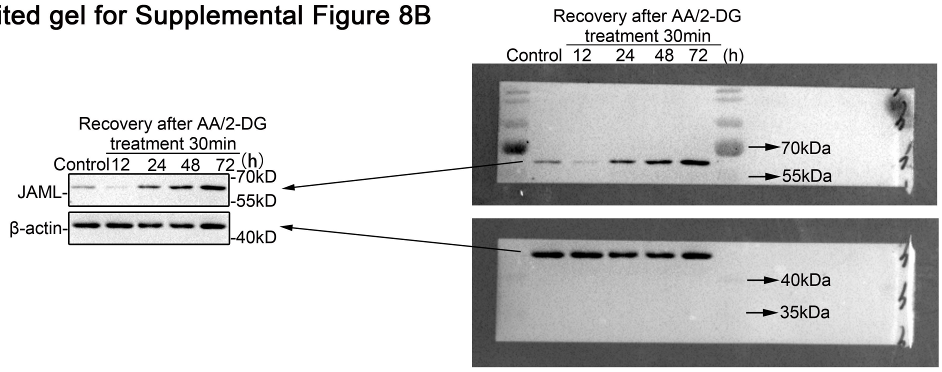

Full unedited gel for Supplemental Figure 8C

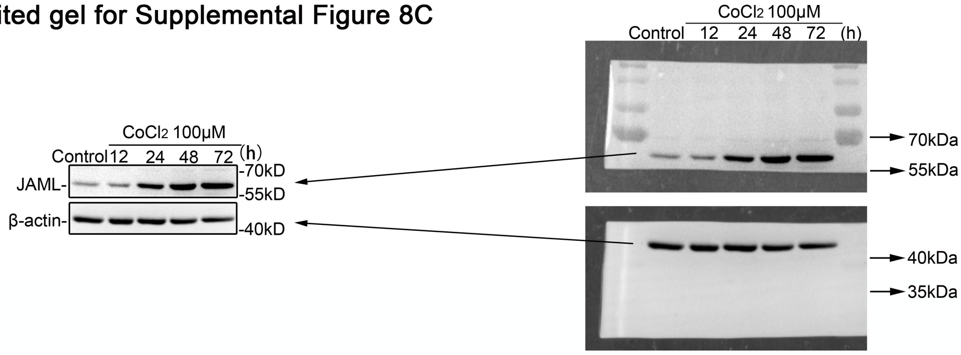

Full unedited gel for Supplemental Figure 8D

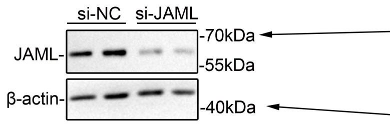

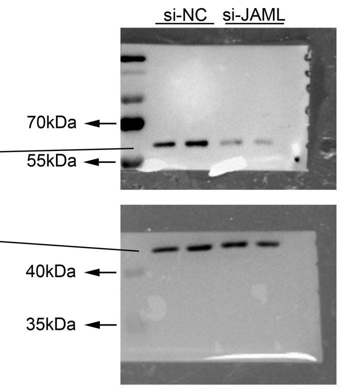

Full unedited gel for Supplemental Figure 8F

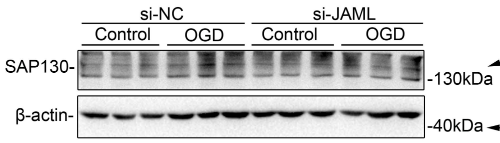

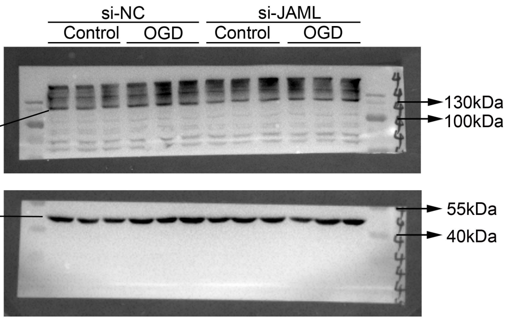

Full unedited gel for Supplemental Figure 8G

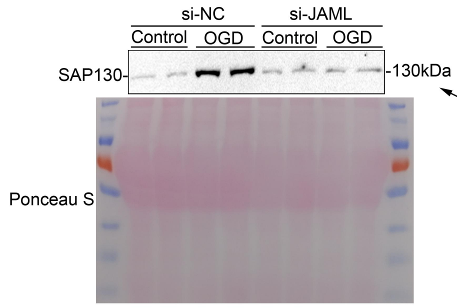

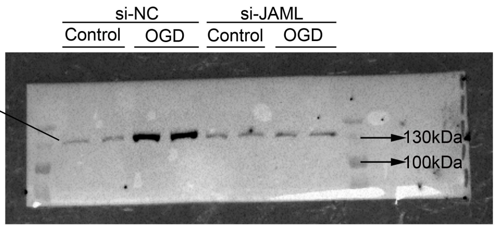

Supplement: Supplemental data [file jciinsight-7-158571-s030.pdf]
